# The rational development of CD5-targeting biepitopic CARs with fully human heavy-chain-only antigen recognition domains

**DOI:** 10.1016/j.ymthe.2021.07.001

**Published:** 2021-07-16

**Authors:** Zhenyu Dai, Wei Mu, Ya Zhao, Xiangyin Jia, Jianwei Liu, Qiaoe Wei, Taochao Tan, Jianfeng Zhou

**Affiliations:** 1 Department of Hematology, Tongji Hospital, Tongji Medical College, Huazhong University of Science and Technology, Wuhan, Hubei 430030, China; 2 Nanjing IASO Biotherapeutics, Nanjing, Jiangsu 210000, China

**Keywords:** fully human antibody, CD5, biepitopic chimeric antigen receptor, heavy-chain-only antigen recognition domains, T cell malignancies, biepitopic antibody, tumor escape, phage display antibody library

## Abstract

T cell malignancies are a group of hematologic cancers with high recurrence and mortality rates. CD5 is highly expressed in ∼85% of T cell malignancies, although normal expression of CD5 is restricted to thymocytes, T cells, and B1 cells. However, CD5 expression on chimeric antigen receptor (CAR)-T cells leads to CAR-T cell fratricide. Once this limitation is overcome, CD5-targeting CAR-T therapy could be an attractive strategy to treat T cell malignancies. Here, we report the selection of novel CD5-targeting fully human heavy-chain variable (FHV_H_) domains for the development of a biepitopic CAR, termed FHV_H_3/V_H_1, containing FHV_H_1 and FHV_H_3, which were validated to bind different epitopes of the CD5 antigen. To prevent fratricide in CD5 CAR-T cells, we optimized the manufacturing procedures of a CRISPR-Cas9-based CD5 knockout (CD5KO) and lentiviral transduction of anti-CD5 CAR. *In vitro* and *in vivo* functional comparisons demonstrated that biepitopic CD5KO FHV_H_3/V_H_1 CAR-T cells exhibited enhanced and longer lasting efficacy; produced moderate levels of cytokine secretion; showed similar specificity profiles as either FHV_H_1, FHV_H_3, or the clinically tested H65; and is therefore suitable for further development.

## Graphical abstract

**Figure:**
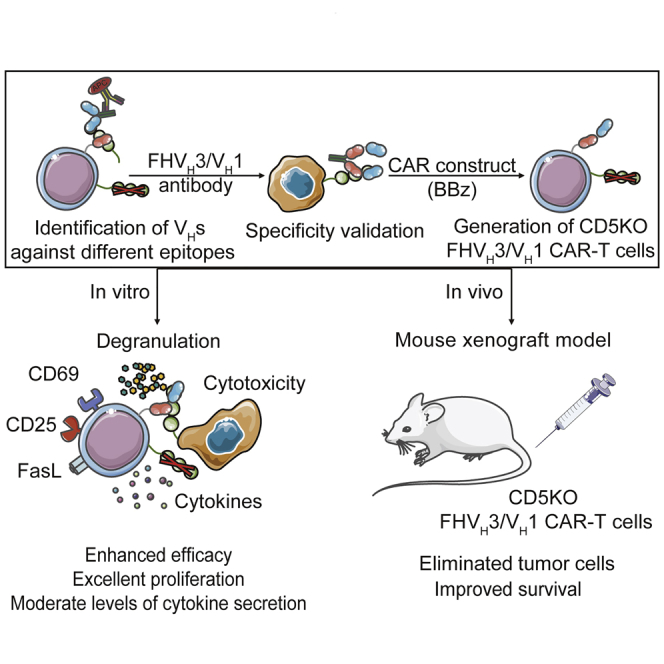

## Teaser

In this article, Jianfeng Zhou and colleagues developed a fully human fratricide-resistant CD5-targeting biepitopic CAR with two V_H_s that bind different epitopes of CD5 antigen, which exhibited enhanced and durable efficacy compared to murine-derived H65 CAR in preclinical study. The newly developed CAR structure may reduce the risk of tumor escape.

## Introduction

In recent years, advances have been made in chimeric antigen receptor T cell (CAR-T) therapy to target B cell malignancies, induce remission, and improve long-term relapse-free survival in patients with B cell leukemia and lymphoma.^1^, ^2^, ^3^, ^4^, ^5^ However, the overall prognosis of refractory or relapse (r/r) T cell malignancies is much poorer compared to B cell malignancies, and salvage chemotherapy regimens remain the best treatment for patients with r/r T cell leukemia or lymphoma.^6^, ^7^, ^8^ Therefore, it is imperative to develop novel effective CAR-T cell therapies to fight T cell malignancies.

CD5 is constitutively expressed on normal T cells and present in ∼85% of T cell malignancies.^9^ It contains three scavenger receptor cysteine-rich (SRCR) domains,^10^, ^11^, ^12^ which act as inhibitory regulators of both T cell receptor (TCR) and B cell receptor (BCR) signaling and serve as a target for evolutionary immunotherapeutic strategies against T cell malignancies.^13^, ^14^, ^15^, ^16^, ^17^, ^18^, ^19^ In addition, CD5 is also frequently expressed in some B cell malignancies, with expression in normal tissues restricted to thymocytes, T cells, and a small subpopulation of B cells (B1 cells).^20^^,^^21^ The data from a phase I clinical trial (ClinicalTrials.gov: NCT03081910) conducted by Baylor College of Medicine demonstrated that CAR-T cells incorporated a murine-derived single-chain variable fragment (scFv) H65 targeting CD5 were safe and had anti-tumor activity in patients with r/r T cell malignancies.^9^ However, the patients who obtained an objective response post-infusion without receiving planned hematopoietic stem cell transplantation relapsed with their latent CD5^+^ malignancy after a few weeks.^9^ Clearly, improvements in the efficacy and persistence of CD5-targeting CAR-T cell therapies for T cell malignancies are urgently needed. The limited lifespan of CAR-T cells in patients may be related to the fratricide of anti-CD5 CAR-T cells and human anti-mouse antibody responses, whereas the application of CD5 knockout (CD5KO) fully human (FH) antibody-derived CAR-T cells may extend the survival and optimal function of CAR-T cells.^22^, ^23^, ^24^, ^25^

Herein, we report the selection of novel FH heavy-chain variable (V_H_) domains and the rational development of a fratricide-resistant, CD5-targeting biepitopic CAR-T therapy. Initially, using a high-quality FH phage display library developed in house containing 8.32 × 10^10^ V_H_ domains, through panning, primary screening, and identification, we obtained some V_H_s that specifically bind to recombinant CD5 protein and cell surface CD5. We hypothesized that the tandem use of V_H_ domains targeting different epitopes could potentially enhance the function of CAR-T cells and minimize the risk of tumor escape due to antigen mutation. Therefore, through competitive binding fluorescence-activated cell sorting (FACS) analysis, FHV_H_1 and FHV_H_3, two V_H_ domains that bind to different epitopes of CD5, were identified. Next, to prolong CAR-T cell persistence, we applied a 4-1BB co-stimulatory domain in the design of the CAR construct that has been reported to possess the capacity to promote CAR-T cell survival^26^^,^^27^ and subsequently optimized the CD5 knockout and lentiviral transduction steps to successfully generate anti-CD5 CAR-T cells that resist fratricide. Furthermore, the functions of degranulation, cytotoxicity, cytokine release, expansion, and persistence of H65, FHV_H_1, FHV_H_3, and biepitopic FHV_H_3/V_H_1 CAR-T cells were confirmed *in vitro* and *in vivo*. FHV_H_3/V_H_1 CAR-T cells exhibited potent and longer lasting efficacy and specificity profiles similar to those of clinically used H65. This biepitopic CAR is currently being prepared for further evaluation and development.

## Results

### Screening and selection of FH CD5-specific V_H_ domains

**Figure 1:**
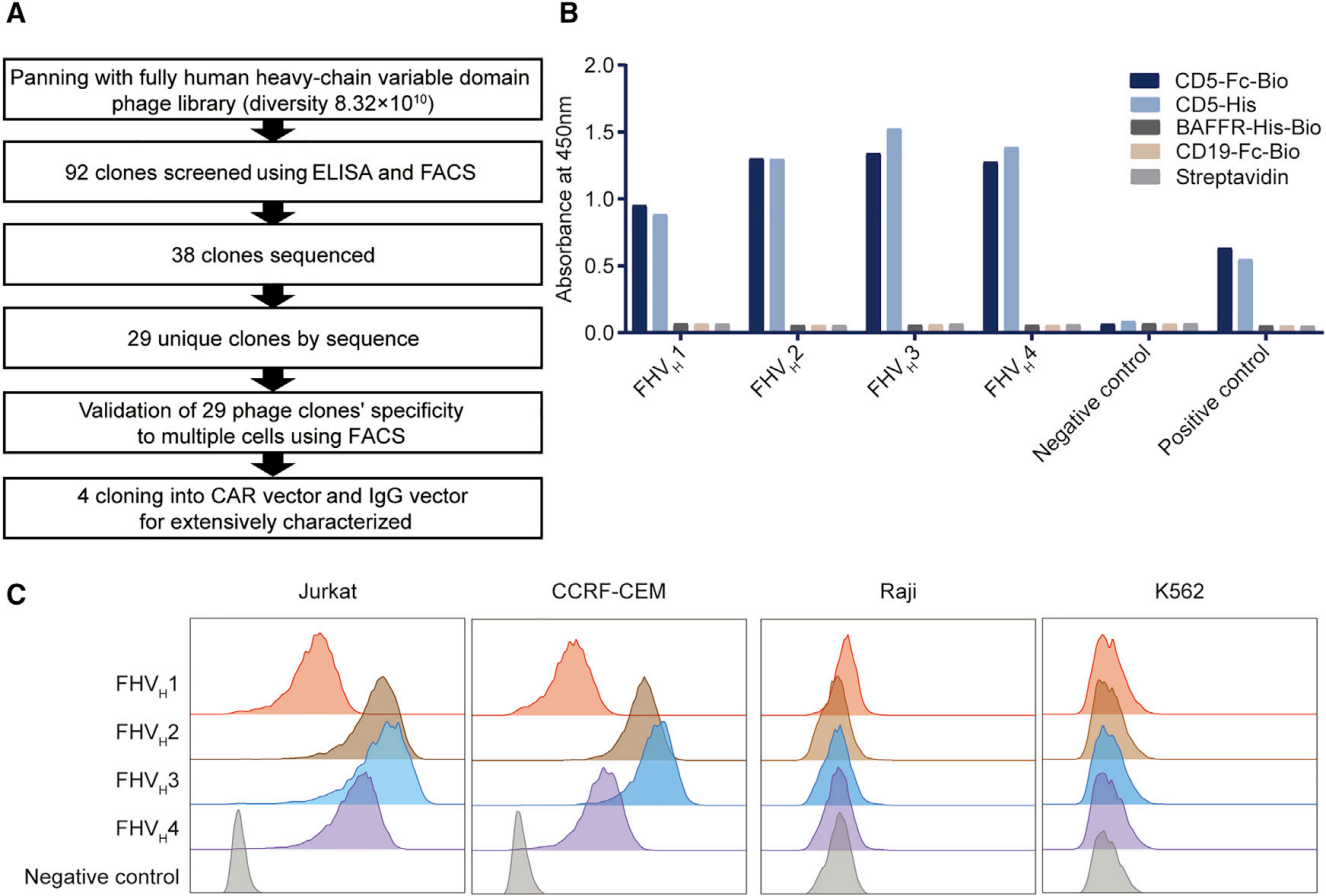
Phage display library screening and selection of fully human (FH) CD5-specific, heavy-chain variable domain (V_H_) (A) Schematic of the CD5-specific V_H_ discovery process. (B) Binding activities of representative phage clones and KO7 (negative control phage) are shown. Binding to different antigens was detected after staining with mouse anti-M13 antibody and horseradish peroxidase (HRP)-goat anti-mouse IgG antibody before reading the optical density at 450 nm. Mouse anti-CD5 and HRP-goat anti-mouse IgG antibodies were used as the positive control. (C) Phage clones FHV_H_1–4 bind to Jurkat and CCRF-CEM (both CD5^+^) cells, but not to Raji and K562 (both CD5^−^) cells. Shown is analyzed using flow cytometry.

A diagram of the screening and selection procedure is shown in Figure 1A. The V_H_ domains were screened from a phage display library comprising both naive and synthetic human V_H_ domains by three rounds of protein panning after enrichment (Table S1). A total of 92 monoclonal antibodies were selected for enzyme-linked immunosorbent assay (ELISA) and validated using CD5^+^ Jurkat and CD5^−^ Raji cells for FACS screening, of which 38 clones specifically bound to CD5 protein, without binding to other control proteins, and the representative clones are shown in Figure 1B. After sequencing, 29 unique clones were identified. The binding specificity of these 29 clones with additional CD5^+/−^ cell lines was further characterized by FACS analysis. Of these, four clones (FHV_H_1–4) were confirmed to bind to another human-derived CD5^+^ cell line (CCRF-CEM; Figure 1C). To further measure their binding affinities to the CD5 antigen, these V_H_ clones, along with H65, were expressed in V_H_/scFv-human immunoglobulin Fc fragment (hFc) or V_H_/scFv-rabbit immunoglobulin Fc fragment (rFc) format and purified. Binding affinity was determined using the Octet96e system. FHV_H_1 (K_D_ = 1.63 × 10^−9^ M), FHV_H_2 (K_D_ = 8.32 × 10^−9^ M), and FHV_H_3 (K_D_ = 1.39 × 10^−9^ M) showed slightly lower but comparable binding affinity to CD5 protein compared with H65 (K_D_ = 6.53 × 10^−10^ M), although FHV_H_4 (K_D_ = 2.47 × 10^−8^ M) exhibited substantially lower affinity than the above clones (Figure S1).

### Identification of V_H_ domains against different epitopes of CD5 and specificity validation of anti-CD5 biepitopic antibodies

**Figure 2:**
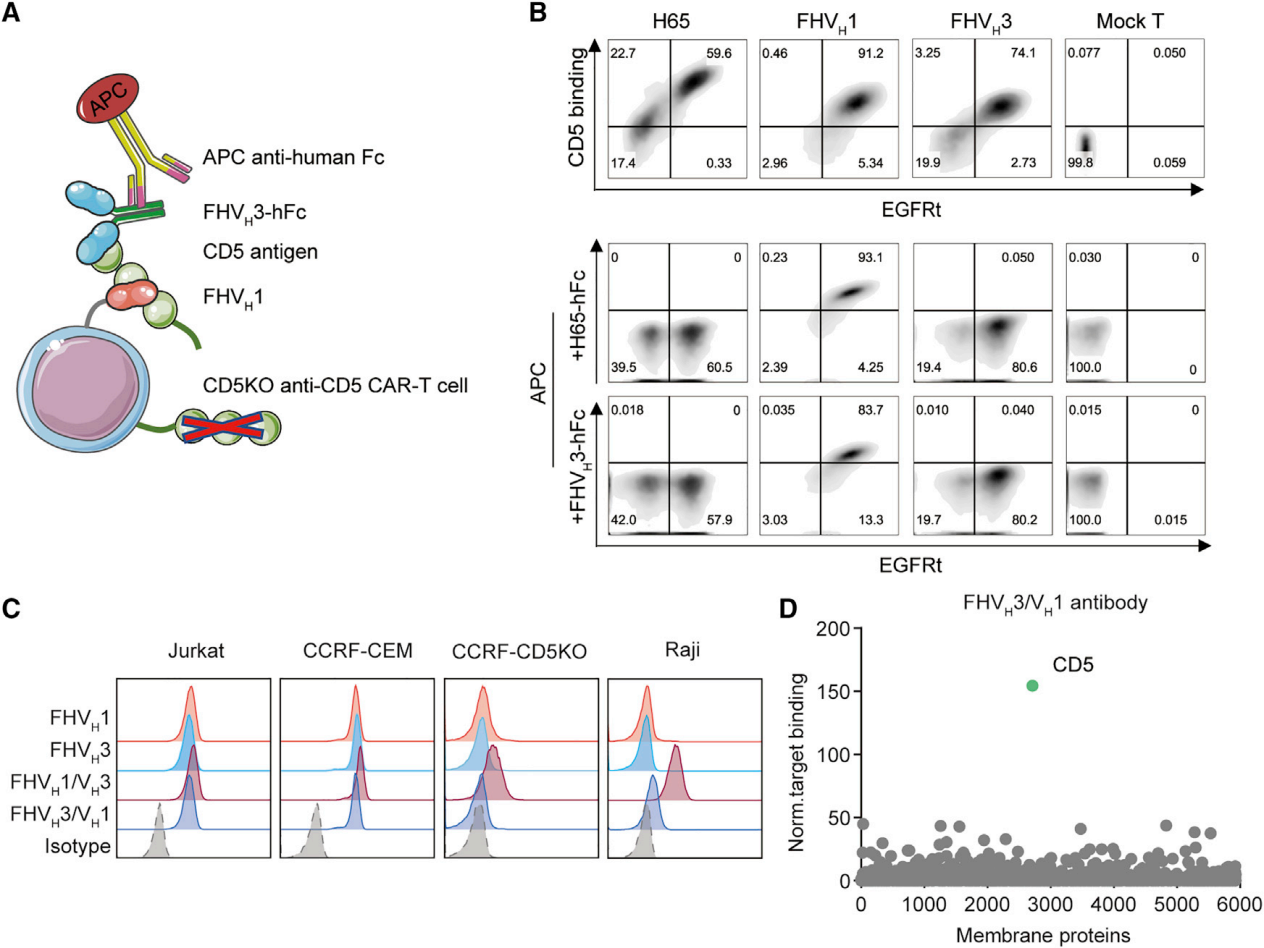
CD5 antigen binding assay and competitive binding analysis of V_H_ and validation of anti-CD5 biepitopic antibodies (A) Schematic diagram of the anti-CD5 V_H_ binding using a competitive fluorescence-activated cell sorting assay. (B) Representative flow cytometry analysis shows that H65, fully human (FH)V_H_1, and FHV_H_3 CAR-T cells all bind to CD5 antigen. FHV_H_3 recognizes the overlapped epitope of CD5 as H65, whereas FHV_H_1 binds different CD5 antigen epitopes with FHV_H_3 and H65. (C) Binding specificities of anti-CD5 biepitopic antibodies are shown. CD5^+^ cell lines (Jurkat and CCRF-CEM) and CD5^−^ cell lines (CCRF-CD5KO and Raji) were stained with isotype control, FHV_H_1-hFc, FHV_H_3-hFc, FHV_H_1/V_H_3-hFc, and FHV_H_3/V_H_1-hFc antibody, respectively, followed by a secondary APC-labeled anti-human IgG, and analyzed using FACS. (D) Binding of FHV_H_3/V_H_1 to QT6 cells individually expressing 5,900 full-length human membrane proteins as determined by flow cytometry is shown.

A diagram of the anti-CD5 V_H_ domains competitive binding assay is shown (Figure 2A). H65, FHV_H_1, and FHV_H_3 CAR-T cells all bind to CD5 antigen, and interestingly, H65-hFc and FHV_H_3-hFc antibodies did not affect the binding of FHV_H_1 CAR-T cells to CD5 antigen, suggesting that FHV_H_1 binds different CD5 antigen epitopes with H65 and FHV_H_3, although FHV_H_3 recognizes the overlapping epitope of CD5 as H65 (Figure 2B). Having demonstrated that FHV_H_1 and FHV_H_3 bind different CD5 antigenic epitopes, we hypothesized that the use of FHV_H_1 and FHV_H_3 in tandem may both enhance the efficacy and reduce the risk of tumor escape caused by antigenic mutations. Before functional validation, we validated the binding specificity of anti-CD5 antibodies to determine whether the tandem use of FHV_H_1 and FHV_H_3 alters their specificity. The results suggested that FHV_H_1, FHV_H_3, and FHV_H_3/V_H_1 antibodies specifically bind CD5^+^ cells (Jurkat and CCRF-CEM), but not CD5^−^ cells, from different tissue sources (Figures 2C and S2). FHV_H_3/V_H_1 antibody was also verified with membrane proteome array (MPA) specificity validation to test for reactivity against approximately 5,900 different membrane protein clones representing more than 90% of the human membrane proteome, with no significant non-specific binding detected (Figure 2D). In addition, the binding affinity of FHV_H_3/V_H_1-hFc (K_D_ = 7.01 × 10^−10^ M) was barely changed compared to that of either FHV_H_1-hFc (K_D_ = 1.63 × 10^−9^ M) or FHV_H_3-hFc (K_D_ = 1.39 × 10^−9^ M; Figure S1). However, the FHV_H_1/V_H_3-hFc antibody showed non-specific binding to CD5^−^ cells (CCRF-CEM-CD5 knockout [CCRF-CD5KO] and Raji) and was excluded from further functional comparisons. Size-exclusion, high-performance liquid chromatography (SEC-HPLC) results suggested that the FHV_H_3/V_H_1-hFc antibody migrated as the major peak (>93%) with a retention time of 11.457 min, although the FHV_H_1/V_H_3-hFc antibody showed four peaks, with the main peak occupying 45.51% of the total peak area with a retention time of 11.948 min (Figure S3), indicating instability of the FHV_H_1/V_H_3-hFc antibody.

### Process optimization of CD5KO and lentiviral transduction eliminate fratricide of anti-CD5 CAR-T cells

**Figure 3:**
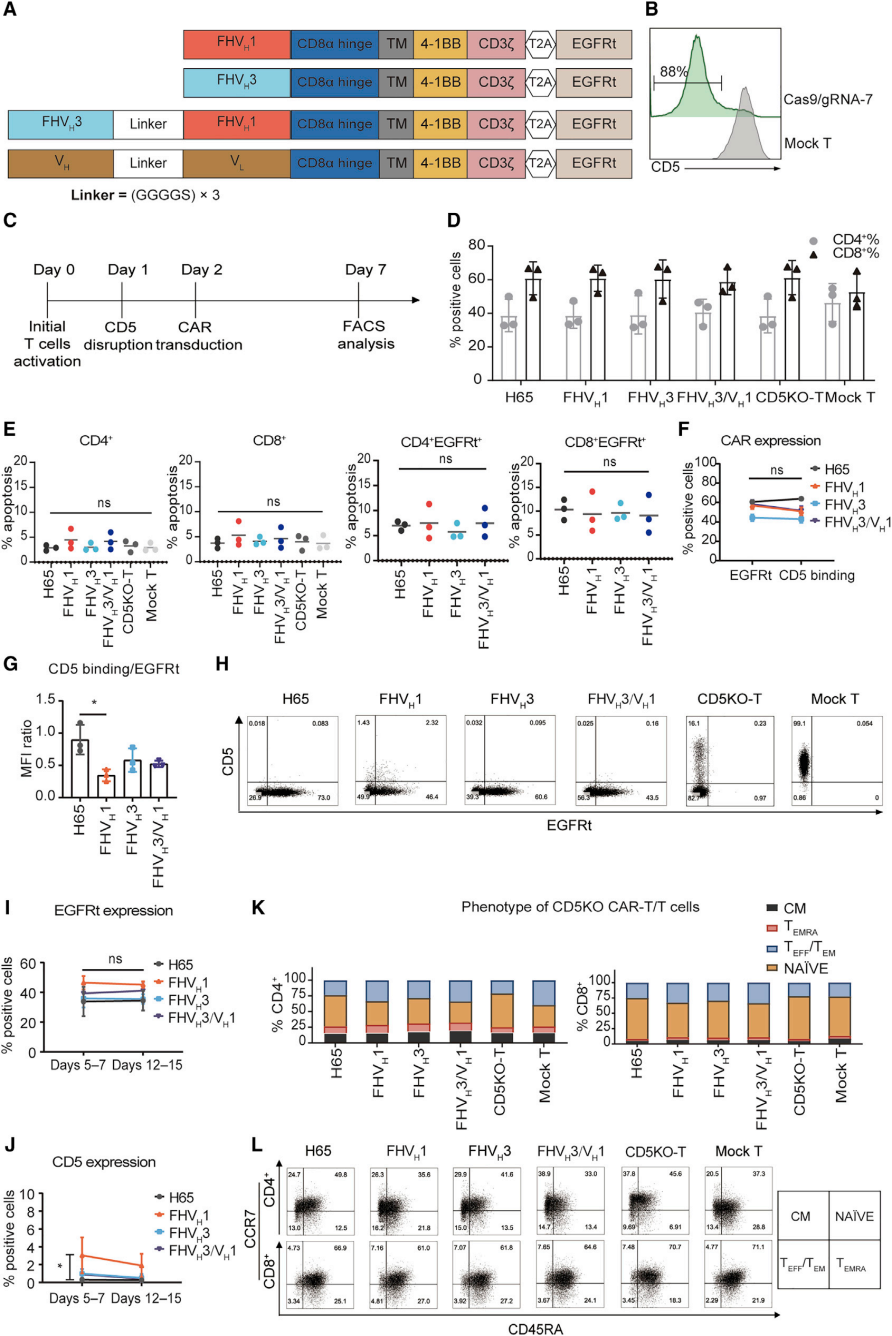
Apoptosis analysis and phenotypic analysis of CD5 knockout anti-CD5 CARs (A) Schematic structures containing the anti-CD5 V_H_, anti-CD5 biepitopic V_H_ domains, or murine-derived H65 scFv. (B) Gene editing efficiency in human primary T cells was determined 3 days after electroporation with Cas9 protein and CD5-specific gRNA-7 by FACS. The numbers indicated the frequency of CD5^−^ cells. (C) Schematic outline of the optimized process for generating CD5KO anti-CD5 CAR-T cells is shown. (D) Proportion of CD4^+^ T cells and CD8^+^ T cells in CAR-T/T cells is shown. The data represent mean ± SD (n = 3). (E) The basal apoptosis of CAR-T/T cells is shown. The four CARs had similar background level of apoptosis as measured by annexin V and PI staining 10 days post-transduction. The data represent mean ± SD (n = 3). ns, not significant (one-way ANOVA). (F) CAR expression on CD5KO-T cells detected by anti-EGFR antibody and recombinant CD5-Fc staining on day 7 is shown. The data represent mean ± SD (n = 3). ns, not significant (two-way ANOVA). (G) Median fluorescence intensity ratios (CD5 antigen binding to EGFRt) of CD5KO-T cells expressing different CARs are shown. The data represent mean ± SD for three donors. ∗p < 0.05 (one-way ANOVA). (H) Representative flow cytometry analysis shows surface expression of CD5 and transduction efficiency of CD5KO anti-CD5 CAR-T/T and mock T cells on day 7. (I) Flow cytometry analysis of EGFRt expression on the surface of CD5 CAR-T cells at different time points is shown. The data represent mean ± SD for three donors. ns, not significant (two-way ANOVA). (J) Flow cytometry analysis of CD5 antigen expression on the surface of anti-CD5 CAR-T cells at different time points is shown. The data represent mean ± SD for three donors. ∗p < 0.05 (two-way ANOVA). (K) Frequency of effector and effector-memory (T_EFF_/T_EM_) (CCR7^−^ CD45RA^−^), effector memory revertant (T_EMRA_) (CCR7^−^ CD45RA^+^), naive (NAÏVE) (CCR7^+^ CD45RA^+^), and central memory (CM) (CCR7^+^ CD45RA^−^) in anti-CD5 CAR-T/T cells assessed using flow cytometry on days 7–10 post-transduction is shown. The data represent the average from three donors. (L) Representative dot plots show the phenotype of activated T cells in (K) after 10 days.

FHV_H_1, FHV_H_3, FHV_H_3/V_H_1, and H65 were incorporated into a second-generation CAR consisting of CD5 antigen recognition domains, a CD8α hinge/transmembrane region, 4-1BB co-stimulatory domain, and intracellular CD3ζ coupled in frame with a truncated epidermal growth factor receptor (EGFRt) through a T2A sequence (Figure 3A). In preliminary studies, we found that the expression of CAR molecules on CD5^+^ CAR-T cells decreased gradually, although the expression of CD5 increased continuously, and apoptotic T cells accounted for the majority of CAR^+^ T cells (CD5^+^ H65 CAR-T cells 53.6% ± 11.1%; n = 3; Figure S4). To address the potential fratricide issue of CAR-T cells, we optimized the procedures of T cell CD5KO and lentiviral transduction of anti-CD5 CAR. Schematic diagrams of 3 different strategies to generate CD5KO anti-CD5 CAR-T cells are shown in Figure S4A. We found that T cells transduced with anti-CD5 CAR lentivirus 24 h after CD5KO showed stable expression of CAR molecules on the cell surface, without CD5 recurrence; expressed relatively low levels of apoptosis (CD5KOLV H65 CAR-T cells 23.8% ± 7.0%; CD5KO24hLV H65 CAR-T cells 10.3% ± 1.4%; n = 3; Figures S4B–S4D); and improved CD5^+^ tumor cell cytolytic capacity (Figure S4E). After electroporation with Cas9 protein and CD5-specific guide RNA (gRNA-7), loss of surface CD5 expression was detected 3 days after electroporation (generally >80%; Figure 3B). Generation of CAR-T cells deficient in CD5 expression was performed as shown in Figure 3C. CD5KO and expression of anti-CD5 CAR did not affect the CD4/CD8 ratio of primary T cells (Figure 3D). Furthermore, apoptosis was not significantly enhanced in any of the CD5KO anti-CD5 CAR-T cells containing 4-1BB co-stimulatory domain compared to either the CD5KO-T cells or mock T cells and also not among the CAR^+^ T cells in either of the four CAR-T cell groups (p > 0.05; n = 3; Figure 3E). This indicates that the loss of CD5 overcomes the unintended fratricide and dysfunction of CAR-T cells. The transduction efficiency of CD5 CARs detected with EGFR antibody was consistent with the CD5 antigen binding rate, and the CD5 antigen binding/EGFRt median fluorescence intensity (MFI) ratio of FHV_H_3/V_H_1 CAR was also not significantly different from that of either the H65, FHV_H_1, or FHV_H_3 CARs (p > 0.05; n = 3; Figures 3F and 3G). FHV_H_1 CAR had a lower CD5 antigen binding/EGFRt MFI ratio than that of the H65 CAR, indicating that the expression degree of FHV_H_1 CAR on the surface of T cells was lower than that of H65 CAR (p < 0.05; n = 3; Figure 3G). The expression of CD5 CARs was easily detected through the expression of EGFRt in primary T cells, and the transduction of activated CD5KO-T cells with lentiviral vectors resulted in efficient CAR expression (Figure 3H). Moreover, CAR expression on CD5KO-T cells remained stable during *in vitro* culture (Figure 3I). The CD5 antigen expression on the surface of anti-CD5 CAR-T cells was analyzed on days 5–7 and 12–15, and, with the exception of FHV_H_1 CAR-T cells, CD5 expression on the surface of H65, FHV_H_3, and FHV_H_3/V_H_1 CAR-T cells was less than 1% on days 12–15 (Figure 3J), in accordance with the requirements for CAR-T cell product manufacturing. The majority of CD5KO anti-CD5 CAR-T cells displayed a naive-like surface phenotype that might have an enhanced capacity for expansion, differentiation, and self-renewal upon antigen stimulation (Figures 3K and 3L).^28^

### CD5KO anti-CD5 biepitopic CAR-T cells exhibited enhanced degranulation and cytotoxicity against malignant T cell lines *in vitro*

**Figure 4:**
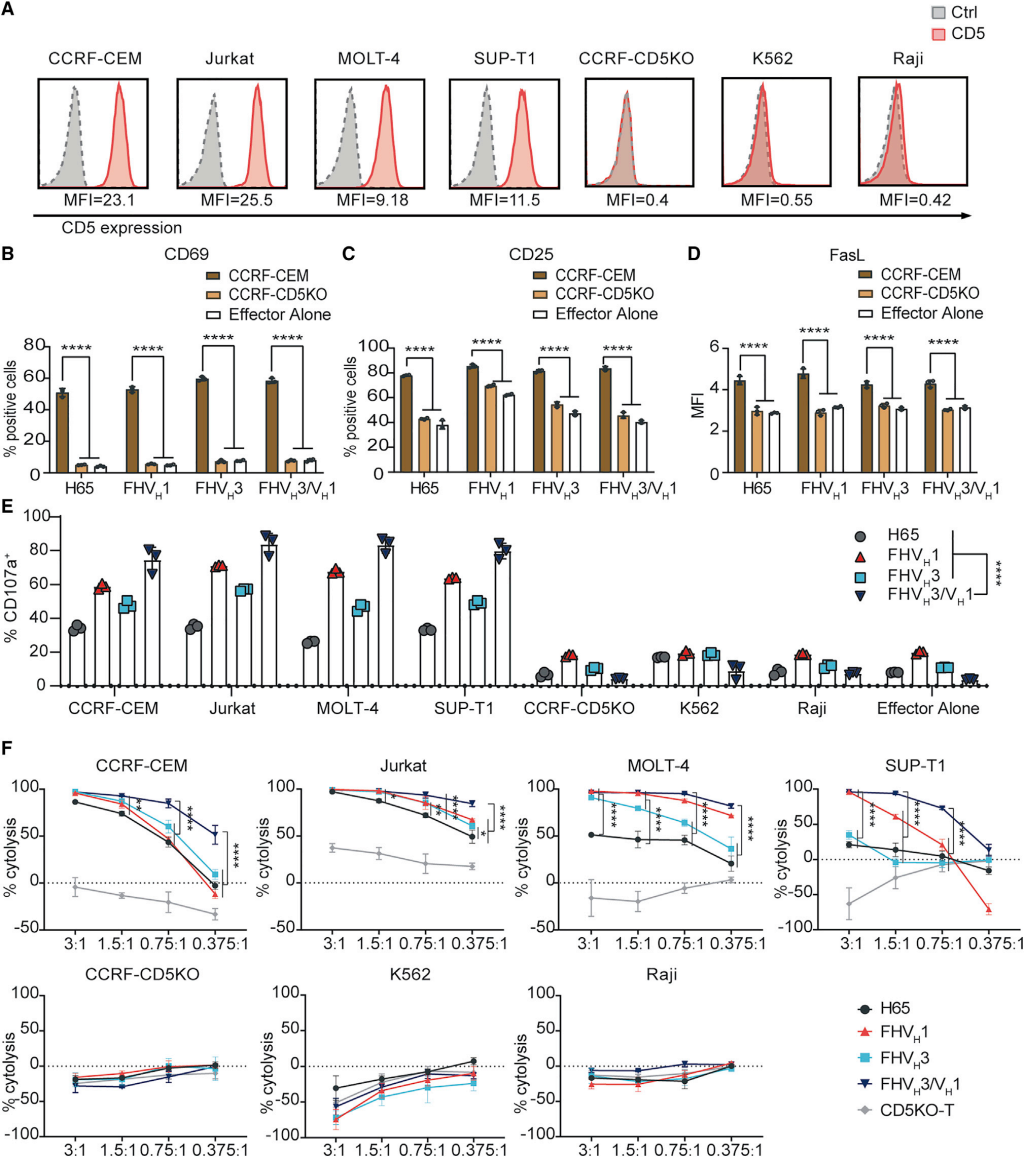
CD5KO anti-CD5 CAR-T cells were compared functionally (A) Surface expression of CD5 (red solid histograms) in T-ALL and T-lymphoma cell lines in comparison with isotype control (dotted line gray histograms) measured using flow cytometry. (B) Expression of the early T cell activation marker CD69 on anti-CD5 CAR-T cells following a 24-h co-incubation with CCRF-CEM or CCRF-CD5KO (gated on CD8^+^ EGFR^+^ cells) is shown. The data represent mean ± SD (n = 3). ∗∗∗∗p < 0.0001 (two-way ANOVA). (C) Expression of the T cell activation marker CD25 on anti-CD5 CAR-T cells following co-incubation as described in (B) is shown (gated on CD8^+^ EGFR^+^ cells). The data represent mean ± SD (n = 3). ∗∗∗∗p < 0.0001 (two-way ANOVA). (D) Median fluorescent intensity of FasL on anti-CD5 CAR-T cells following co-incubation as described in (B) is shown (gated on CD8^+^ EGFR^+^ cells). The data represent mean ± SD (n = 3). ∗∗∗∗p < 0.0001 (two-way ANOVA). (E) The degranulation assay of four CARs is shown. CAR-T cells were stimulated with target cells expressing different CD5 antigen densities. The CD5 antigen-specific increase in CD107a was assessed as a measure of degranulation. The data represent mean ± SD for three donors. ∗∗∗∗p < 0.0001 (two-way ANOVA). (F) Anti-CD5 CAR-T cells selectively kill CD5^+^ tumor cells. All CARs lyse CD5^+^ target cells in a dose-dependent manner. The killing ability of CAR-T/T cells for CD5^+^ cell lines was determined by luciferase-based cytotoxicity assay after 24 h incubated with target cells at different E:T ratios. The data indicate mean ± SD from three co-cultures. ∗p < 0.05, ∗∗p < 0.01, and ∗∗∗∗p < 0.0001 (two-way ANOVA).

CD5 is widely expressed in T cell malignancies. We found that CCRF-CEM and Jurkat cell lines were highly CD5 expressing, MOLT-4 and SUP-T1 were moderately CD5 expressing, and CCRF-CD5KO, K562, and Raji were CD5^−^ (Figure 4A). CD5KO anti-CD5 CAR-T cells expressed significantly higher levels of the T cell activation markers CD69 and CD25 after co-culture with CCRF-CEM, but not after co-culture with CCRF-CD5KO (p < 0.0001; Figures 4B and 4C). FHV_H_1 CAR-T cells stimulated by CD5^+^ target cells CCRF-CEM showed a slightly higher median fluorescence intensity of Fas ligand (FasL) than either FHV_H_3/V_H_1, FHV_H_3, or H65 (p < 0.05; Figure 4D), although T cells showed both enhanced killing activity against tumor cells and an increased risk of activation-induced cell death (AICD) as a result of augmented FasL expression.^29^^,^^30^

Degranulation was a prerequisite for CAR-T cell perforin-granzyme-mediated killing, CD5KO anti-CD5 CAR-T cells upregulated CD107a (a surrogate marker for degranulation) expression in a CD5-specific manner, and FHV_H_3/V_H_1 CAR-T cells exhibited significantly higher degranulation compared to either FHV_H_1, FHV_H_3, or H65 after CD5 antigen stimulation (p < 0.0001; Figure 4E). Next, the cytotoxicity of CD5KO anti-CD5 CAR-T cells was assessed using a luciferase-based assay. CD5KO anti-CD5 CAR-T cells selectively killed tumor cells expressing different CD5 antigen densities in a dose-dependent manner. CD5KO anti-CD5 CAR-T cells showed robust cytotoxicity against CD5^+^ tumor cell lines at the indicated effector to target ratios, whereas they were not cytotoxic against the CD5^−^ cell lines CCRF-CD5KO, K562, and Raji (Figure 4F), demonstrating that the cytolysis was CD5 specific. In particular, CD5KO anti-CD5 biepitopic CAR-T cells exhibited greater cytotoxicity *in vitro* than either FHV_H_1, FHV_H_3, or H65, especially when co-incubated with malignant T cell lines with moderate expression of CD5 antigen at relatively low effector to target (E:T) ratios (Figure 4F). Similarly, compared to either FHV_H_1, FHV_H_3, or H65 CAR-T cells, biepitopic FHV_H_3/V_H_1 CAR-T cells exhibited higher levels of degranulation and greater cytotoxicity after *in vitro* co-incubation with K562-CD5 L1–3 stable cell lines with relatively low levels of CD5 antigen expression (Figure S5).

**Figure 5:**
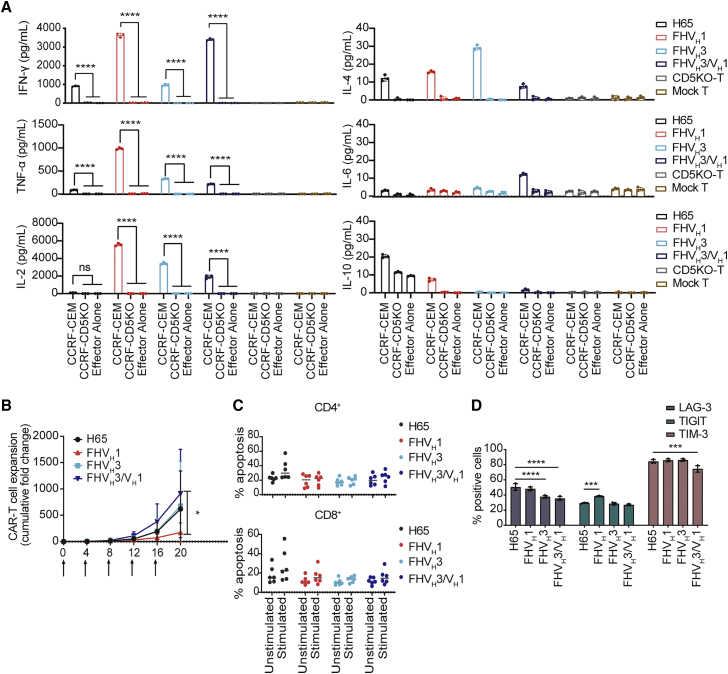
Cytokine secretion and repeated stimulation assay of CD5-targeting CAR-T cells *in vitro* (A) Quantification of cytokines (TNF-α, IFN-γ, IL-2, IL-4, IL-6, and IL-10) from the supernatant after CAR-T/T cells were co-cultured with CCRF-CEM and CCRF-CD5KO at an E:T ratio of 1:1 for 24 h. The results are displayed as mean ± SD (n = 3). ∗∗∗∗p < 0.0001 (two-way ANOVA). (B) Repeated antigen stimulation expansion assay is shown: anti-CD5 CAR-T cells were co-cultured with mitomycin-C-treated CCRF-CEM cells. Every 4 days, CAR-T cells were counted and then the same number of CAR-T cells were re-mixed with mitomycin-C-treated CCRF-CEM cells again. The data represent mean ± SD from three donors. ∗p < 0.05 (two-way ANOVA). (C) Apoptosis assay of anti-CD5 CAR-T cells was performed before and after twice stimulated by mitomycin-C-treated CCRF-CEM cells. The results are displayed as mean ± SD (n = 6). (D) Anti-CD5 CAR-T cells were stained with antibodies against CD8, EGFR, LAG-3, TIGIT, and TIM-3, respectively, before and after 7 stimulations by CCRF-CEM cells. The results are displayed as mean ± SD (n = 3). ∗∗∗p < 0.001 and ∗∗∗∗p < 0.0001 (two-way ANOVA).

Moreover, all four types of CAR-T cells produced the pro-inflammatory cytokines tumor necrosis factor alpha (TNF-α) and interferon (IFN)-γ in response to CD5^+^ CCRF-CEM cells (Figure 5A). Except for H65, respectively FHV_H_1, FHV_H_3, and FHV_H_3/V_H_1 CAR-T cells showed elevated levels of interleukin-2 (IL-2) release when co-incubated with CCRF-CEM cells. Notably, FHV_H_3/V_H_1 CAR-T cells secreted lower levels of CD5 antigen-specific TNF-α and IL-2 than both FHV_H_1 and FHV_H_3 CAR-T cells. Therefore, cytolytic degranulation between CAR-T cells and target cells is assumed to be one of the main mechanisms leading to better anti-tumor activity of biepitopic FHV_H_3/V_H_1 CAR-T cells. It also suggested that the tandem CAR construct of FHV_H_1 and FHV_H_3 may not exacerbate the risk of cytokine release syndrome (CRS) although possessing enhanced tumor-killing capacity. Collectively, FHV_H_3/V_H_1 CAR-T cells demonstrated potent and specific tumor-killing activity against CD5^+^ cells.

### CD5KO anti-CD5 biepitopic CAR-T cells exhibited an excellent expansion after repeated antigen stimulation *in vitro*

The expansion capacity of CAR-T cells in an environment of continuous exposure to target antigen is critical for eradicating the large tumor burden and maintaining sustained remission. To this end, we sought to assess the expansion potential, expression of exhaustion markers, and apoptosis levels of CAR-T cells *in vitro* over multiple cycles of antigen stimulation. FHV_H_3/V_H_1 CAR-T cells exhibited significantly higher proliferation than FHV_H_1 (p < 0.05) and slightly higher proliferation than FHV_H_3 and H65 (no significant difference; p > 0.05) after five rounds of mitomycin-C-treated CCRF-CEM cell stimulation (Figure 5B). The apoptosis levels of anti-CD5 CAR-T cells were not significantly different in both CD4^+^ and CD8^+^ subsets before and after stimulation with mitomycin-C-treated CCRF-CEM cells (Figure 5C). Furthermore, FHV_H_3/V_H_1 CAR-T cells expressed lower levels of LAG-3 and TIM-3 after the 7th stimulation in CCRF-CEM cells than H65 CAR-T cells, and T cell immunoreceptor with Ig and ITIM domains (TIGIT) expression levels of FHV_H_1 CAR-T cells were higher than those of H65 CAR-T cells (p < 0.001; Figure 5D).

### CD5KO anti-CD5 biepitopic CAR-T cells demonstrated superior antitumor activity *in vivo*

**Figure 6:**
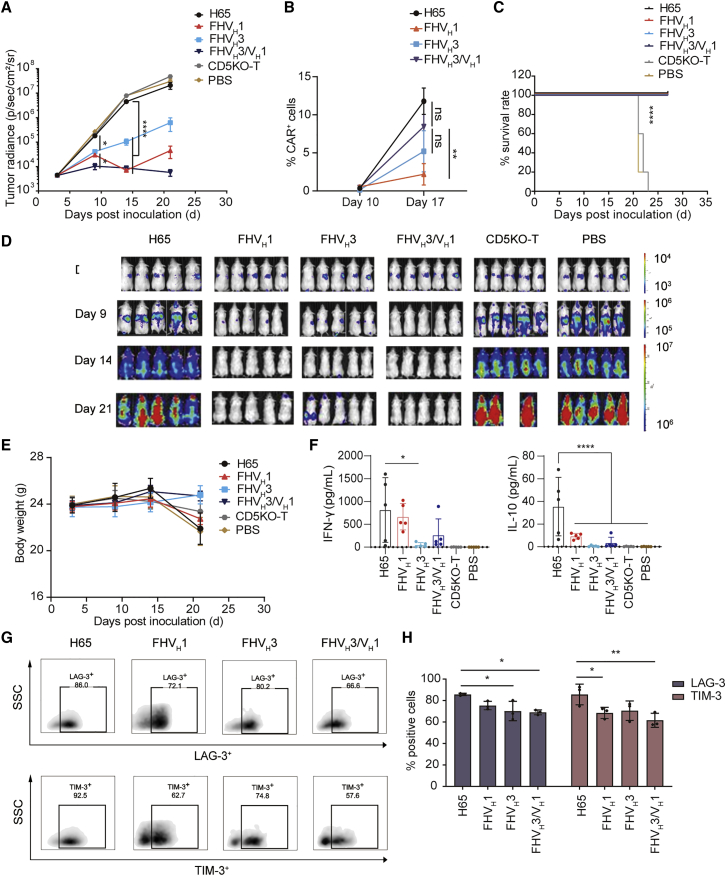
The *in vivo* antitumor activity of CD5-targeting CAR-T cells in the tumor model established by CCRF-CEM (A) Average radiance quantification of each treatment group measured at the indicated time points. The results are displayed as mean ± SEM (n = 5). ∗p < 0.05 and ∗∗∗∗p < 0.0001 (two-way ANOVA). (B) Relative frequency of CD5KO anti-CD5 CAR-T cells (CD45^+^ EGFR^+^) in peripheral blood of mice on days 10 and 17 is shown, respectively (n = 3). ∗∗p < 0.01 (two-way ANOVA). (C) Overall Kaplan-Meier survival curve is shown. Survival curves were compared using the log rank test. Mice treated with CAR-T cells showed significantly increased survival (∗∗∗∗p < 0.0001) compared with those of CD5KO-T and PBS-treated groups. (D) Growth and staging of the tumor monitored by bioluminescence imaging are shown. (E) Body weight curve is shown. The results are displayed as mean ± SEM (n = 5). (F) Concentration of IFN-γ and IL-10 in the serum of indicated groups collected on day 17 is shown. The results are displayed as mean ± SD (n = 5). ∗p < 0.05 and ∗∗∗∗p < 0.0001 (one-way ANOVA). (G) Representative flow cytometry analysis shows the proportion of LAG-3^+^ and TIM-3^+^ cells in CAR-T cells in the peripheral blood of NCG mice collected on day 24. (H) Quantification and statistical analysis of the results in (G) are shown. The results are displayed as mean ± SD (n = 3). ∗p < 0.05 and ∗∗p < 0.01 (two-way ANOVA).

After completing the functional validation of CD5 CAR-T cells *in vitro*, we established a mouse tumor model of T cell acute lymphoblastic leukemia (T-ALL) by tail intravenous injection of CCRF-CEM-ffLuc cells to verify the *in vivo* efficacy of CD5 CAR-T cells. We tested the ability of CD5 CAR-T cells administered on days 4 and 7 post-engraftment to suppress leukemia progression. The results showed that FHV_H_3/V_H_1 CAR-T cells cleared T-ALL cells earlier than FHV_H_1 and FHV_H_3 CAR-T cells and maintained a longer remission than FHV_H_1 and FHV_H_3 CAR-T cells, whereas the same dose of H65 CAR-T cells only had a weak antitumor effect (Figure 6A). The relative percentage of CD45^+^ EGFR^+^ T cells in the peripheral blood on days 10 and 17 was detected using flow cytometry. The frequency of FHV_H_3/V_H_1 CAR-T cells was significantly higher than that of FHV_H_1 CAR-T cells on day 17 (p < 0.01), although there was no significant difference in the frequency of either H65 or FHV_H_3 CAR-T cells (p > 0.05; Figure 6B). The relative percentages of H65, FHV_H_3, and FHV_H_3/V_H_1 CAR-T cells was significantly higher on day 17 than on day 10 (p < 0.01), whereas FHV_H_1 CAR-T cells did not show significant expansion (p > 0.05; Figure 6B). All CARs were effective in prolonging the survival of tumor-bearing mice (Figure 6C). Importantly, in contrast to the mice receiving either H65 CAR-T or CD5KO-T cells, which showed massive leukemic burden by bioluminescence imaging (BLI), the mice treated with FHV_H_3/V_H_1 CAR-T cells were practically leukemia free by day 21, whereas the mice administered FHV_H_1 and FHV_H_3 CAR-T cells appeared to have tumor recurrence (Figure 6D). There was no significant difference in body weight of these mice among six groups (Figure 6E).

Subsequently, we performed the quantitative determination of Th1/Th2 cytokines in the serum of PBS- or CAR-T/T-treated mice on day 17 after CCRF-CEM cell infusion. We detected high levels of IFN-γ and IL-10 in the serum of mice in the H65 CAR-T cell treatment group collected on day 17 (Figure 6F). TNF-α, IL-2, IL-4, and IL-6 were barely detectable in the serum of all groups of mice collected on day 17, and TNF-α, IFN-γ, IL-2, IL-4, IL-6, and IL-10 were undetectable in the serum of all groups of mice collected on day 10 (data not shown). In addition, the results of flow cytometry analysis showed that FHV_H_3/V_H_1 CAR-T cells in the peripheral blood of tumor-bearing mice collected on day 24 exhibited lower levels of LAG-3 and TIM-3 than H65 CAR-T cells (Figures 6G and 6H).

**Figure 7:**
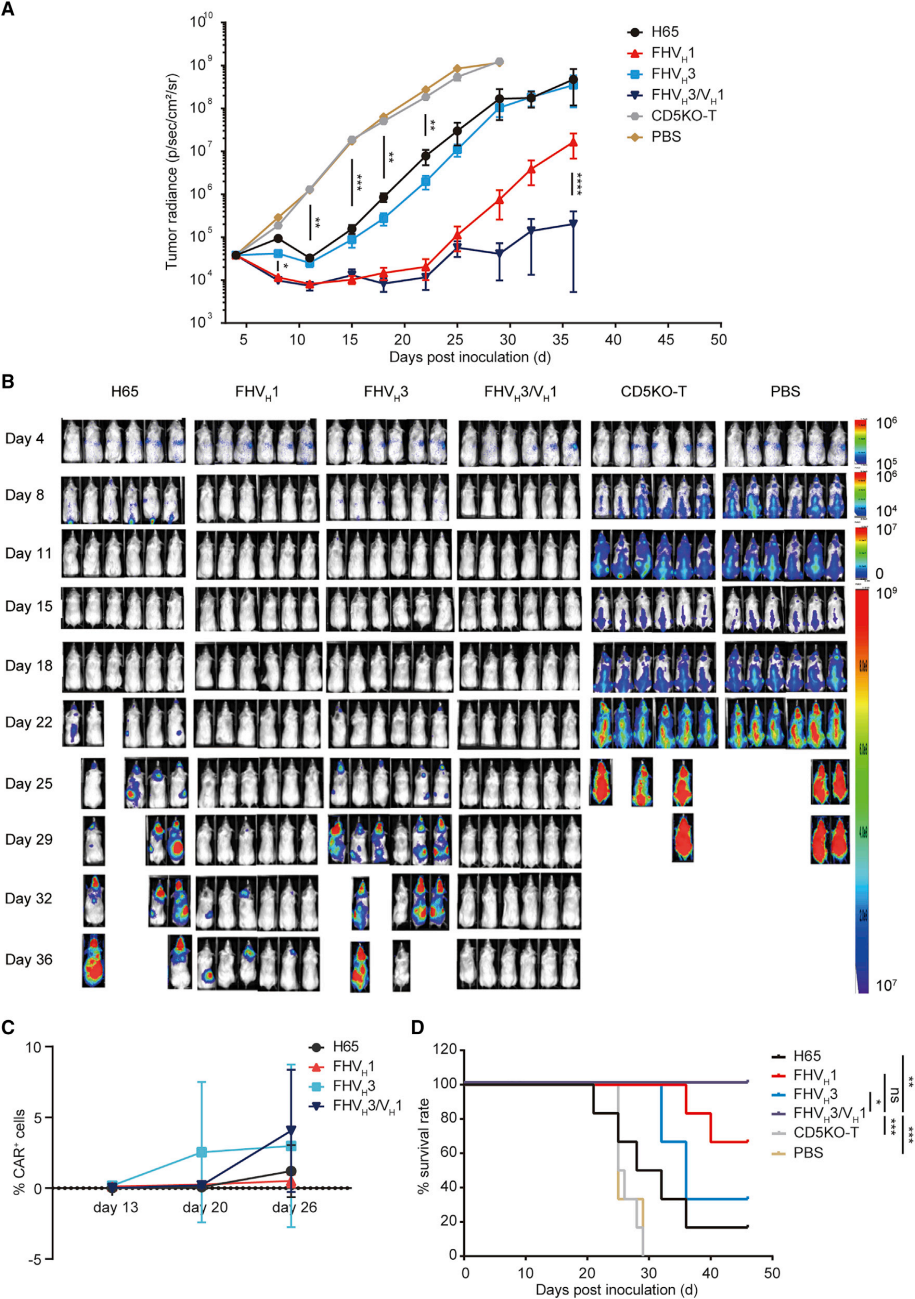
The *in vivo* antitumor activity of CD5-targeting CAR-T cells in the tumor model established by SUP-T1 (A) Mouse tumor burden of each treatment group at the indicated time points. The results are displayed as mean ± SEM (n = 6). ∗p < 0.05, ∗∗p < 0.01, ∗∗∗p < 0.001, and ∗∗∗∗p < 0.0001 (two-way ANOVA). (B) Growth and staging of the tumor monitored by bioluminescence imaging are shown. (C) Relative frequency of CD5KO anti-CD5 CAR-T cells (CD45^+^ EGFR^+^) in peripheral blood of mice on day 13, day 20, and day 26, respectively (n = 6). (D) Overall Kaplan-Meier survival curve is shown. ∗p < 0.05, ∗∗p < 0.01, and ∗∗∗p < 0.001 (log rank test).

In the xenograft mouse model established by SUP-T1 cells with moderate CD5 expression, after infusion of 2 × 10^6^ CAR^+^ T cells per mouse, H65 and FHV_H_3 CAR-T cells temporarily inhibited and controlled cancer progression but failed to eradicate neoplastic cells (Figures 7A and 7B). In contrast, FHV_H_3/V_H_1 and FHV_H_1 CAR-T cells eliminated neoplastic cells in mice at an early stage, and FHV_H_3/V_H_1 CAR-T cells significantly prolonged the suppression of leukemia compared with FHV_H_1 CAR-T cells (p < 0.0001; Figures 7A and 7B). To evaluate the expansion and persistence of CAR^+^ T cells, the percentage of CD45^+^ EGFR^+^ cells in the peripheral blood of mice was determined using flow cytometry on days 13, 20, and 26. The results demonstrated that FHV_H_3/V_H_1 CAR-T cells on day 26 showed significant expansion compared to day 13 and day 20 (p < 0.05), although the frequency changes of CAR-T cells in other groups were not significantly different (p > 0.05; Figure 7C). Moreover, the survival time of the tumor-bearing mice in either the FHV_H_3/V_H_1 or FHV_H_1 CAR-T-cell-treated groups was significantly longer than that in either the H65 or FHV_H_3 CAR-T-cell-treated groups (Figure 7D).

## Discussion

As shown in a recent clinical trial, CAR-T therapy targeting CD5 is an attractive strategy in patients with r/r CD5^+^ T cell malignancies without resulting in a life-threatening immunodeficiency.^9^ However, preliminary data from the clinical trial using murine-derived H65 CAR-T therapy indicated a good safety profile but modest and non-persistent efficacy (44% of patients obtained an objective response).^9^ Self-activation and fratricide caused by expression of CD5 antigen on the anti-CD5 CAR-T cells may contribute to poor persistence, which in turn leads to CD5^+^ tumor recurrence. Therefore, to fully utilize the potential of anti-CD5 CAR-T cell therapy and address the concerns of human anti-mouse immune responses, we developed a fratricide-resistant biepitopic CAR-T therapy derived from original FH CD5 V_H_s. Recently, several preclinical studies of FH single-domain, antibody-derived CAR-T cell therapies have demonstrated *in vitro* and *in vivo* functions similar to those of murine scFv-derived benchmarks, such as CD33-targeting V_H_ (CAR33V_H_) and B cell maturation antigen (BCMA)-targeting V_H_ (FHV_H_33).^31^^,^^32^ CARs derived from FH heavy-chain-only binding domains have significant advantages over scFv-binding domains. Heavy-chain-only binding domains simplify the structural design of CARs, and the smaller size of V_H_ domains has potential steric advantages over the larger scFv domains in accessing cryptic antigenic epitopes.^33^^,^^34^ Through three rounds of CD5 antigen panning, we successfully enriched and obtained phage V_H_ clones that were validated by FACS and ELISA to bind specifically to the CD5 antigen. Using a competitive FACS assay and specificity validation assay, we obtained two FH V_H_ domains (FHV_H_1 and FHV_H_3) that specifically bind to different epitopes of CD5. Biepitopic FHV_H_3/V_H_1 antibody was verified with MPA specificity validation and binding test to cell lines of different tissue origins to further ensure specificity and safety. Notably, we found that the structural arrangement of V_H_ domains is one of the factors affecting the stability of biepitopic antibodies.

Subsequently, to further compare the function of candidate CAR molecules and to eliminate the adverse effects of fratricide on CAR-T cells, we performed optimization of CD5 knockout and lentiviral transduction of primary T cells. After optimization, the CRISPR-Cas9-based CD5 knockout efficiency of T cells was above 80% and CAR expression rates of fratricide-resistant CD5 CAR-T cells remained stable. As a negative regulator of antigen-receptor-mediated signaling in thymocytes and T cells, CD5 knockout in mice did not induce changes in populations of T and B lymphocytes compared to control mice.^12^^,^^35^^,^^36^ Recently, Alotaibi et al.^37^ reported that functionally blocking CD5 signaling resulted in enhanced antitumor immunity and elevated T cell activation. Chun et al.^22^ also demonstrated that CD5 knockout enhances the anti-tumor activity of CAR-T cells by enhancement of CAR-mediated activation and proliferation. In our study, CD5 knockout did not alter the CD4/CD8 ratio and phenotype of T cells and was able to prevent activation-induced cell death and dysfunction of CAR-T cells due to autoantigen stimulation.

By applying the optimized procedures, we performed a functional comparison of tandem V_H_ CARs using either linkers of different sizes, flexibilities, and amino acid compositions or V_H_ domains connected in different orders (i.e., V_H_1-V_H_3 or V_H_3-V_H_1). These tandem CAR constructs are shown in Figure S6A. All tandem CARs could be successfully expressed on the surface of T cells; however, FHV_H_1 (EAAAK) × 1 V_H_3 CAR-T cells were shown to have poorer ability to eliminate CD5^+^ T cells than other tandem CARs (p < 0.01; Figures S6B and S6C). The functional comparison revealed that the tandem V_H_ CAR construct with a long flexible (G4S)3-linker, with lower levels of CD107a background, had relatively robust degranulation and cytotoxicity against high or moderate CD5-expressing target cells, higher levels of TNF-α release, and comparable IFN-γ release, compared to other constructs (Figures S6D–S6F). This indicates the importance of linker selection and connected order of tandem V_H_ domains for the structural stability and functional performance of CARs.

Afterward, we compared the *in vitro* and *in vivo* functions of biepitopic FHV_H_3/V_H_1 CAR-T cells with H65, FHV_H_1, and FHV_H_3 CAR-T cells targeting a single epitope. As expected, FHV_H_3/V_H_1 CAR-T cells exhibited stronger CD5 antigen-specific degranulation and killing ability than either H65, FHV_H_1, or FHV_H_3 CAR-T cells, especially for tumor cells with moderate expression of CD5 antigen. Remarkably, FHV_H_3/V_H_1 CAR-T cells showed excellent expansion and persistence and did not express higher levels of exhaustion markers and activation-induced cell death than the other groups of CAR-T cells in both *in vivo* and *in vitro* target antigen stimulation assays. In addition, despite the significant functional enhancement, FHV_H_3/V_H_1 CAR-T cells maintained relatively modest cytokine secretion, which might not exacerbate CRS and neurotoxicity in patients after infusion.

To further strengthen our hypothesis that tandem V_H_ domains targeting different epitopes could potentially enhance the function of CAR-T cells, wild-type CD5-expressing and mutated CD5-expressing K562 cell lines were generated and utilized for the localization of functional epitopes recognized by anti-CD5 CAR-T cells (Figures S7A–S7C). FHV_H_3/V_H_1 and FHV_H_1 CAR-T cells showed increased degranulation after stimulation by K562-CD5-DX23 but neither H65 nor FHV_H_3 CAR-T cells (Figure S7D), indicating that H65 and FHV_H_3 CAR-T cells could only bind the membrane-distal region (epitope near D1), whereas FHV_H_3/V_H_1 and FHV_H_1 CAR-T cells could recognize the membrane-proximal region (epitope near D2). Chimeric TCR^+^ CTL targeting membrane-proximal CD22 epitope was reported to show potent degranulation and cytotoxicity compared to those binding to membrane-distal epitope.^38^ In our study, FHV_H_1 CAR, targeting membrane-proximal epitope, exhibited stronger cytotoxicity and cytokine release compared to the membrane-distal epitope targeting CAR (H65 and FHV_H_3). In addition, stimulation with K562-CD5-D123 resulted in higher levels of FHV_H_3/V_H_1 CAR-T cell degranulation compared to stimulation with K562-CD5-DX23 (p < 0.0001; Figure S7D), reflecting a significant synergistic effect. Xu et al.^39^ previously reported the development of a biepitopic llamas-derived BCMA-targeting CAR-T therapy and applied this system to achieve a better objective response rate, complete remission rate, and lower relapse rate than monovalent BCMA CAR-T therapy in clinical trials.^40^ With a similar conceptual basis and excellent preclinical results, we speculate that FHV_H_3/V_H_1 CAR-T cells could also demonstrate favorable efficacy and safety in future clinical studies. Moreover, due to its superior efficacy and potentially lower immunogenicity, the FHV_H_3/V_H_1 CAR may also be of interest when used for “off-the-shelf” universal anti-CD5 CAR-T/CAR-NK therapy.

CAR-T therapy targeting CD5 has good clinical application prospects beyond just in the treatment of T cell malignancies; with high expression on a subset of B cell malignancies, it can also be used to treat B cell malignancies, such as mantle cell lymphoma, diffuse large B cell lymphoma, and chronic lymphocytic leukemia or small-cell lymphocytic lymphoma.^41^, ^42^, ^43^ Furthermore, application of CAR-T therapy to target multiple antigens simultaneously is an attractive strategy for treatment and prevention of antigen-loss relapses, and V_H_ binding domains furthermore ease the design of multispecific CARs.^44^, ^45^, ^46^, ^47^ The use of two V_H_ domains can simplify the design of bispecific CAR constructs capable of recognizing different antigens compared to the design utilizing two scFv domains. Therefore, FHV_H_1 and FHV_H_3 are suitable for further use in the design of bispecific or even multispecific CAR structures to solve critical problems in current cancer drug development, such as clonal heterogeneity and antigen escape.

## Materials and methods

### Cell lines

CD5^+^ cell lines, Jurkat (acute T cell leukemia), CCRF-CEM (acute T lymphoblastic leukemia), MOLT-4 (acute T lymphoblastic leukemia), SUP-T1 (T cell lymphoblastic lymphoma), and the CD5^−^ cell lines, K562 (chronic myelogenous leukemia), Raji (Burkitt’s lymphoma), and NALM-6 (acute B-lymphocytic leukemia), were cultured in RPMI-1640 medium containing 10% fetal bovine serum (FBS) (Thermo Fisher Scientific, Waltham, MA, USA). QT6 (fibrosarcoma), HCT-116 (colorectal carcinoma), HEPG2 (hepatocellular carcinoma), MDA-MB-468 (breast adenocarcinoma), OVCAR3 (ovarian adenocarcinoma), NCI-H460 (large cell lung cancer), 293CT/293T (embryonic kidney), KATO III (gastric carcinoma), and PANC-1 (pancreatic epithelioid carcinoma) were cultured in DMEM (Corning, Corning, NY, USA) medium containing 10% FBS (Thermo Fisher Scientific). All cell lines were verified before use. CCRF-CD5KO is a human CD5 knockout cell line generated from CCRF-CEM by CRISPR-Cas9. K562-CD5 L1–5 are stable cell lines with varying degrees of expression of human CD5 molecules, as determined by flow cytometry sorting. K562-CD5-D123 expresses all three CD5 SRCR domains, named D1, D2, and D3. K562-CD5-DX23 deletion mutant lacks D1, and D1 and D2 were removed from K562-CD5-DXX3.

### Screening for FH anti-CD5 V_H_ domains

A FH heavy-chain-only phage display antibody library (IMARS; Nanjing IASO Biotherapeutics, Nanjing, China) was used to generate anti-CD5 V_H_ domains by optimal protein panning. In brief, three rounds of bead panning were performed using CD5-hFc-Bio as the target antigen and CD19-hFc-Bio as the counterpart. After three rounds of panning, the CD5-specific phages were enriched. The phage clones were first tested for their ability to bind to recombinant CD5 via ELISAs using plates coated with CD5-hFc-Bio (Kactus Biosystems, Shanghai, China)/CD5-His (ACRO Biosystems, Newark, DE, USA) and streptavidin or control antigen CD19-hFc-Bio (ACRO Biosystems)/BAFFR-His-Bio (Kactus Biosystems) and streptavidin (Pierce, Rockford, IL, USA). KO7 (M13KO7 helper phage; Invitrogen, Waltham, MA, USA) served as a negative control. The specificity of these clones to Jurkat/Raji was evaluated by flow cytometry. After sequencing, to validate the binding specificity of unique clones, their binding affinities to multiple CD5^+^ (Jurkat and CCRF-CEM) and CD5^−^ (Raji and K562) cell lines were further confirmed using flow cytometry. Clones with good specificity to both recombinant CD5 protein and cell lines were constructed using CARs and immunoglobulin G (IgG) proteins for further analysis. The anti-CD5 biepitopic CAR and IgG protein vector constructs involved FHV_H_1 and FHV_H_3. In the IgG protein vector constructs, the two clones were linked by a flexible (G4S)3-linker with either the V_H_1 at the N terminus (V_H_1-V_H_3) or the V_H_3 at the N terminus (V_H_3-V_H_1). Tandem V_H_ CARs used either linkers of different sizes, flexibilities, and amino acid compositions or V_H_ domains connected in different orders (i.e., V_H_1-V_H_3 or V_H_3-V_H_1).

### Generation of CD5KO anti-CD5 CAR-T cells

FH anti-CD5 V_H_ domains and the control H65 scFv were grafted into a second-generation CAR with a CD8α hinge/transmembrane region, 4-1BB co-stimulatory domain, and intracellular CD3ζ, with the CD5 CAR gene linked to a EGFRt by a T2A sequence for further translational and clinical research.

Lentivirus was generated using Lipo3000 (Invitrogen) by transient transduction of Lenti-X293T cells with psPAX2 and pMD2.G packaging plasmids. The viral supernatants were collected at either 48 h or 72 h after transduction, filtered, concentrated, aliquoted, and stored at −80°C.

Human donor peripheral blood leukocytes from healthy donors were used for *in vitro* and *in vivo* CAR-T/T functional validation. The protocol was approved by the Institutional Review Board of Tongji Hospital, Tongji Medical College, Huazhong University of Science and Technology. Appropriate informed consent was obtained from all donors before specimen collection, following the Declaration of Helsinki. Peripheral blood mononuclear cells (PBMCs) were isolated from the collected blood leukocytes via density gradient centrifugation using Ficoll-Paque Plus (GE Healthcare, Boston, MA, USA). CD3^+^ T cells from PBMCs were purified with CD3 microbeads (Miltenyi Biotec, Bergisch Gladbach, Germany) according to the manufacturer’s instructions. Then, T cells were cultured in X-VIVO 15 medium (Lonza, Basel, Switzerland) supplemented with 10% FBS (Thermo Fisher Scientific), 100 U/mL IL-2 (Sigma-Aldrich, St. Louis, MO, USA), and activated with Dynabeads Human T-Activator CD3/CD28 (Thermo Fisher Scientific). After 1 day of activation, the CD5 gene was genomically disrupted in primary human T cells using a Celetrix electroporation system (Celetrix, Manassas, VA, USA) following the manufacturer’s instructions.^48^ Then, CD5KO-T cells were transduced with lentivirus at a multiplicity of infection of 2–5 1 day later. Following electroporation, T cells were incubated in X-VIVO 15 medium supplemented with 10% FBS in the presence of IL-7 (40 ng/mL; Novoprotein Scientific, Summit, NJ, USA) and IL-15 (50 ng/mL; Novoprotein Scientific).

### CD5 antigen binding assay and competitive binding FACS analysis of VH domains

For the antigen binding detection, CD5KO CAR-T and mock T cells (1 × 10^6^) were harvested and incubated for 1 h at 4°C with CD5-hFc-Bio (0.4 μg/mL; Kactus Biosystems), washed twice, and then incubated with allophycocyanin (APC)-conjugated streptavidin (BioLegend, San Diego, CA, USA) before subjected to flow cytometry analysis.

For the competitive binding assay, CD5KO CAR-T and mock T cells (1 × 10^6^) were co-cultured with a pre-mixed solution of FHV_H_3-hFc (10 μg/mL) or H65-hFc (10 μg/mL) and CD5-His (0.4 μg/mL; ACROBiosystems) for 1 h at 4°C, respectively. After washing twice, the cells were incubated with APC-conjugated goat anti-human IgG Fc antibody (Jackson ImmunoResearch Laboratories, West Grove, PA, USA), and the cells were analyzed using flow cytometry.

### Specificity validation of anti-CD5 biepitopic antibodies

For specificity analysis of biepitopic antibodies, FHV_H_1/V_H_3 and FHV_H_3/V_H_1 were engineered into full-length antibody formats with human or rabbit IgG1 Fc regions. CD5^+^ cell lines (1 × 10^6^) and CD5^−^ cell lines (1 × 10^6^) were harvested and incubated for 1 h at 4°C with either isotype control, FHV_H_1, FHV_H_3, FHV_H_1/V_H_3, or FHV_H_3/V_H_1 antibody (10 μg/mL); washed twice; incubated with either APC-conjugated anti-human IgG antibody (polyclonal; Jackson ImmunoResearch Laboratories) or phycoerythrin (PE)-conjugated anti-rabbit IgG antibody (clone: Poly4064; BioLegend); and then analyzed using flow cytometry.

### MPA specificity validation

Specificity testing of FHV_H_3/V_H_1 using MPA was accomplished by Integral Molecular (Philadelphia, PA, USA). The MPA comprises approximately 5,900 different membrane protein clones, representing more than 90% of the human membrane proteome. The binding activity across the protein library was measured on an Intellicyt iQue (Essen BioScience, Ann Arbor, MI, USA), whereas each target identified with MPA screening was reaffirmed in a second flow cytometry experiment after continuous dilutions of FHV_H_3/V_H_1 antibody, as previously described.^49^

### Flow cytometry-based assays

The cell lines were stained with APC-conjugated mouse anti-human CD5 antibody (clone: UCHT2; BD PharMingen, San Diego, CA, USA) and isotype antibody (clone: MOPC-21; BioLegend) to determine the CD5 antigen expression level.

PE or APC-conjugated EGFR antibody (clone: AY13), fluorescein isothiocyanate (FITC)-conjugated CD8 (clone: SK1), PE/cyanine7-conjugated CD4 (clone: A161A1), BV421-conjugated FasL (clone: NOK-1), BV421-conjugated CCR7 (clone: G043H7), APC-conjugated CD45RA (clone: HI100), FITC-conjugated CD8 (clone: SK1), BV421-conjugated TIM-3 (clone: F38-2E2), APC-conjugated LAG-3 (clone: 7H2C65), PE/cyanine7-conjugated TIGIT (clone: A15153G), and FITC-conjugated CD45 (clone: HI30) antibodies were all purchased from BioLegend. CAR-T cells (4 × 10^5^ CAR^+^) were co-incubated with equal tumor cells for 24 h and then the activation markers CD69 and CD25 were detected with BV421-conjugated CD69 antibody (clone: FN50; BioLegend) and FITC-conjugated CD25 antibody (clone: BC96; BioLegend) and the activation-induced surface expression of FasL was also detected. For T cell exhaustion detection, CAR-T cells (1 × 10^5^ CAR^+^) were co-incubated with equal CCRF-CEM cells for 48 h and then equal amounts of CCRF-CEM cells were added again and incubated for another 48 h and repeated seven times in total. After that, the exhaustion markers TIM-3, LAG-3, and TIGIT were detected with BV421-conjugated TIM-3, APC-conjugated LAG-3, and PE/cyanine7-conjugated TIGIT antibodies. The data were acquired with MACS Quant Analyzer 10 (Miltenyi Biotec) and analyzed with FlowJo software version 10 (Tree Star, Ashland, OR, USA).

### Apoptosis assays

Apoptotic cell death was analyzed by annexin V/phosphatidylinositol (PI) staining, and CD5KO CAR-T/T cells (5 × 10^5^) were harvested and stained with FITC Annexin V Apoptosis Detection Kit with PI (BioLegend) following the manufacturer’s instructions and then subjected to flow cytometry analysis to detect apoptosis.

### Degranulation

For the degranulation assay, CD5KO CAR-T cells were co-incubated with different target cells for 4 h in the presence of 1:50 PE/cyanine7-conjugated CD107a antibody (clone H4A3; BioLegend) and 1:500 monensin (BioLegend), and CD107a was detected using flow cytometry.

### Cytolysis assays

To determine the cytotoxicity of CD5KO anti-CD5 CAR-T cells against CD5^+^ cell lines, Jurkat, CCRF-CEM, MOLT-4, SUP-T1, CCRF-CD5KO, K562, and Raji cell lines were stably transduced with firefly luciferase via lentivirus, and the monoclonal stably ffLuc-expressing cell lines were generated by limiting dilution. For CAR-T cell killing assays, target cells (2 × 10^4^) were plated in U-bottom 96-well plates in triplicate with CD5KO anti-CD5 CAR^+^ T/T cells at specified E:T ratios and incubated for 24 h. Luciferase assay was performed using the Steady-Glo Luciferase assay system (Promega, Madison, WI, USA) according to the manufacturer’s instructions.

### CBA-based, cytokine-releasing assays

Cytokine-releasing assays were performed by co-incubating 2 × 10^5^ CAR^+^ T/T cells with 4 × 10^5^ target cells at a 1:2 ratio. After further culture for 24 h, the supernatants were collected for cytokine level measurement using a Cytometric Bead Array (CBA) Human Th1/Th2 Cytokine Kit II (BD Biosciences) according to the manufacturer’s instructions. The quantitative determination of cytokines in the serum of PBS- or CAR-T/T-treated mice was also performed using CBA.

### Repeat antigen stimulation expansion

For the repeat antigen stimulation expansion assay, on day 0, CCRF-CEM cells were plated in 6-well plates treated with mitomycin C at a final concentration of 1 μg/mL. On day 1, mitomycin-C-treated CCRF-CEM cells (3 × 10^5^) were washed six times with PBS and then mixed with 3 × 10^5^ viable CAR-T cells in 24-well plates with X-VIVO 15 medium supplemented with IL-2. On day 4, new CCRF-CEM cells were treated as on day 0, viable CAR-T cells were counted, and 3 × 10^5^ CAR-T cells from the 24-well plates that expanded were re-mixed with 1 × 10^5^ mitomycin-C-treated CCRF-CEM cells as on day 1. This process was repeated five times. Fold expansion after each stimulation was calculated as (viable CAR-T cells on day 4)/(3 × 10^5^), whereas the cumulative expansion was calculated by the following equation: expansion folds = (fold expansion_n_) × (fold expansion_n+1_).

### Mouse xenograft models

Animal experiments were accomplished by GemPharmatech (Nanjing, China). The protocol and procedures involving the care and use of animals in this study were reviewed and approved by the Institutional Animal Care and Use Committee (IACUC) of GemPharmatech before the experiments began. The animals were handled in accordance with the regulations of the Association for Assessment and Accreditation of Laboratory Animal Care (AAALAC). 6-week-old female NOD-Prkdc^em26Cd52^Il2rg^em26Cd22^/Nju (NCG) mice were engrafted with 1 × 10^6^ CCRF-CEM-firefly luciferase cells via tail injection on day 0. Then, the mice were treated by infusion with 2 × 10^6^ and 1 × 10^6^ CAR^+^ T cells via tail injection on days 4 and 7 (n = 5 for each group). In the cancer model established by SUP-T1, 6-week-old female NCG mice were engrafted with 1 × 10^6^ SUP-T1-firefly luciferase cells via tail injection on day 0. Then, the mice were treated by infusion with 2 × 10^6^ CAR^+^ T cells via tail injection on day 5 (n = 6 for each group). The leukemic burden was evaluated using bioluminescence imaging, and body mass and survival were monitored.

### Antibody affinity measurement

The binding affinity of FHV_H_1, FHV_H_3, FHV_H_3/V_H_1, and H65 antibodies to CD5 was measured using the Octet96e system (ForteBio, Menlo Park, CA, USA). In brief, anti-CD5 antibodies were diluted to 20 μg/mL with loading buffer and loaded at ∼0.8 nM onto the biosensors. After a 60-s equilibration phase, the binding kinetics of the CD5 antigen were monitored at multiple antigen concentrations (12.5–400 nM). Each concentration was tested for 160 s of association and 300 s of disassociation. The binding kinetics were analyzed using a 1:1 binding site model (Biacore X100 version 2.0; Cytiva, Marlborough, MA, USA).

### SEC-HPLC assays

The SEC-HPLC analysis was performed using an Alliance HPLC Waters 2695 Separation Module attached to a Waters UV detector (Milford, MA, USA). Samples were analyzed with a TSK 3000SWxl column (5 μm; 300 × 7.5 × 300 mm). Each sample (30 μL) was injected, and separation was performed at a flow rate of 0.8 mL/min. The mobile phase consisted of 300 mM NaCl and 50 mM sodium phosphate at pH 6.8. The total run time was 20 min, and UV detection was performed at 280 nm. Empower 3 software (Waters) was used for the data evaluation.

### Intracellular cytokine staining assays

Intracellular cytokine staining assays were performed by co-incubating 1 × 10^5^ CD5KO CAR^+^ T/T cells with 5 × 10^5^ indicated target cells at a 1:1 ratio; brefeldin A (BioLegend) and monensin (BioLegend) were added 1 h after plating according to the manufacturer’s instructions. After further culture for 4 h, cells were incubated with antibodies for surface markers and permeabilized for 20 min using BD FACS Permeabilizing Solution 2, followed by staining with PE-conjugated TNF-α (clone: MAb11; BioLegend) and PE/cyanine7-conjugated IFN-γ (clone: 4S.B3; BioLegend) antibodies.

### Graphs and statistical analysis

Graphs and data analyses were performed using GraphPad Prism Software version 8.3.0. Some of these graphs were obtained and modified from Servier Medical Art. Unless otherwise stated, all data are representative of at least three independent experiments. All data are presented as mean ± SD except for mouse tumor radiance quantification and body weight data shown as mean ± SEM. Significant differences were analyzed by one-way analysis of variance, two-way analysis of variance, or log rank test. p values are represented as either not significant (ns), ∗p < 0.05, ∗∗p < 0.01, ∗∗∗p < 0.001, or ∗∗∗∗p < 0.0001.

### Data availability statement

The data that support the findings of this study are available from the corresponding authors upon reasonable request.

## Supplemental information

Document S1. Figures S1–S7 and Table S1

Document S2. Article plus supplemental information

## References

[1] Neelapu S.S., Locke F.L., Bartlett N.L., Lekakis L.J., Miklos D.B., Jacobson C.A., Braunschweig I., Oluwole O.O., Siddiqi T., Lin Y. (2017). Axicabtagene ciloleucel CAR T-cell therapy in refractory large B-cell lymphoma. N. Engl. J. Med..

[2] Jacobson C.A. (2019). CD19 chimeric antigen receptor therapy for refractory aggressive B-cell lymphoma. J. Clin. Oncol..

[3] Hirayama A.V., Gauthier J., Hay K.A., Voutsinas J.M., Wu Q., Gooley T., Li D., Cherian S., Chen X., Pender B.S. (2019). The response to lymphodepletion impacts PFS in aggressive non-Hodgkin lymphoma patients treated with CD19 CAR T cells. Blood.

[4] Davila M.L., Riviere I., Wang X., Bartido S., Park J., Curran K., Chung S.S., Stefanski J., Borquez-Ojeda O., Olszewska M. (2014). Efficacy and toxicity management of 19-28z CAR T cell therapy in B cell acute lymphoblastic leukemia. Sci. Transl. Med..

[5] Gill S., Frey N.V., Hexner E.O., Lacey S.F., Melenhorst J.J., Byrd J.C., Metzger S., Marcus T., Gladney W., Marcucci K. (2017). CD19 CAR-T cells combined with ibrutinib to induce complete remission in CLL. J. Clin. Oncol..

[6] Vose J., Armitage J., Weisenburger D., International T-Cell Lymphoma Project (2008). International peripheral T-cell and natural killer/T-cell lymphoma study: pathology findings and clinical outcomes. J. Clin. Oncol..

[7] Winter S.S., Dunsmore K.P., Devidas M., Wood B.L., Esiashvili N., Chen Z., Eisenberg N., Briegel N., Hayashi R.J., Gastier-Foster J.M. (2018). Improved survival for children and young adults with T-lineage acute lymphoblastic leukemia: results from the Children’s Oncology Group AALL0434 methotrexate randomization. J. Clin. Oncol..

[8] Jain N., Lamb A.V., O’Brien S., Ravandi F., Konopleva M., Jabbour E., Zuo Z., Jorgensen J., Lin P., Pierce S. (2016). Early T-cell precursor acute lymphoblastic leukemia/lymphoma (ETP-ALL/LBL) in adolescents and adults: a high-risk subtype. Blood.

[9] Hill L.C., Rouce R.H., Smith T.S., Yang L., Srinivasan M., Zhang H., Perconti S., Mehta B., Dakhova O., Randall J. (2019). Safety and anti-tumor activity of CD5 CAR T-cells in patients with relapsed/refractory T-cell malignancies. Blood.

[10] Tabbekh M., Mokrani-Hammani M., Bismuth G., Mami-Chouaib F. (2013). T-cell modulatory properties of CD5 and its role in antitumor immune responses. OncoImmunology.

[11] Huang H.J., Jones N.H., Strominger J.L., Herzenberg L.A. (1987). Molecular cloning of Ly-1, a membrane glycoprotein of mouse T lymphocytes and a subset of B cells: molecular homology to its human counterpart Leu-1/T1 (CD5). Proc. Natl. Acad. Sci. USA.

[12] Perez-Villar J.J., Whitney G.S., Bowen M.A., Hewgill D.H., Aruffo A.A., Kanner S.B. (1999). CD5 negatively regulates the T-cell antigen receptor signal transduction pathway: involvement of SH2-containing phosphotyrosine phosphatase SHP-1. Mol. Cell. Biol..

[13] Leonard J.E., Johnson D.E., Shawler D.L., Dillman R.O. (1988). Inhibition of human T-cell tumor growth by T101-ricin A-chain in an athymic mouse model. Cancer Res..

[14] LeMaistre C.F., Rosen S., Frankel A., Kornfeld S., Saria E., Meneghetti C., Drajesk J., Fishwild D., Scannon P., Byers V. (1991). Phase I trial of H65-RTA immunoconjugate in patients with cutaneous T-cell lymphoma. Blood.

[15] Mamonkin M., Rouce R.H., Tashiro H., Brenner M.K. (2015). A T-cell-directed chimeric antigen receptor for the selective treatment of T-cell malignancies. Blood.

[16] Mamonkin M., Mukherjee M., Srinivasan M., Sharma S., Gomes-Silva D., Mo F., Krenciute G., Orange J.S., Brenner M.K. (2018). Reversible transgene expression reduces fratricide and permits 4-1BB costimulation of CAR T cells directed to T-cell malignancies. Cancer Immunol. Res..

[17] Xu Y., Liu Q., Zhong M., Wang Z., Chen Z., Zhang Y., Xing H., Tian Z., Tang K., Liao X. (2019). 2B4 costimulatory domain enhancing cytotoxic ability of anti-CD5 chimeric antigen receptor engineered natural killer cells against T cell malignancies. J. Hematol. Oncol..

[18] Brossard C., Semichon M., Trautmann A., Bismuth G. (2003). CD5 inhibits signaling at the immunological synapse without impairing its formation. J. Immunol..

[19] Freitas C.M.T., Johnson D.K., Weber K.S. (2018). T cell calcium signaling regulation by the co-receptor CD5. Int. J. Mol. Sci..

[20] Jones N.H., Clabby M.L., Dialynas D.P., Huang H.J., Herzenberg L.A., Strominger J.L. (1986). Isolation of complementary DNA clones encoding the human lymphocyte glycoprotein T1/Leu-1. Nature.

[21] Berland R., Wortis H.H. (2002). Origins and functions of B-1 cells with notes on the role of CD5. Annu. Rev. Immunol..

[22] Chun I., Kim K.H., Chiang Y.-H., Xie W., Lee Y.G.G., Pajarillo R., Rotolo A., Shestova O., Hong S.J., Abdel-Mohsen M. (2020). CRISPR-Cas9 knock out of CD5 enhances the anti-tumor activity of chimeric antigen receptor T cells. Blood.

[23] Raikar S.S., Fleischer L.C., Moot R., Fedanov A., Paik N.Y., Knight K.A., Doering C.B., Spencer H.T. (2017). Development of chimeric antigen receptors targeting T-cell malignancies using two structurally different anti-CD5 antigen binding domains in NK and CRISPR-edited T cell lines. OncoImmunology.

[24] Lamers C.H.J., Willemsen R., van Elzakker P., van Steenbergen-Langeveld S., Broertjes M., Oosterwijk-Wakka J., Oosterwijk E., Sleijfer S., Debets R., Gratama J.W. (2011). Immune responses to transgene and retroviral vector in patients treated with ex vivo-engineered T cells. Blood.

[25] Turtle C.J., Hanafi L.-A., Berger C., Gooley T.A., Cherian S., Hudecek M., Sommermeyer D., Melville K., Pender B., Budiarto T.M. (2016). CD19 CAR-T cells of defined CD4+:CD8+ composition in adult B cell ALL patients. J. Clin. Invest..

[26] Philipson B.I., O’Connor R.S., May M.J., June C.H., Albelda S.M., Milone M.C. (2020). 4-1BB costimulation promotes CAR T cell survival through noncanonical NF-κB signaling. Sci. Signal..

[27] Long A.H., Haso W.M., Shern J.F., Wanhainen K.M., Murgai M., Ingaramo M., Smith J.P., Walker A.J., Kohler M.E., Venkateshwara V.R. (2015). 4-1BB costimulation ameliorates T cell exhaustion induced by tonic signaling of chimeric antigen receptors. Nat. Med..

[28] Cieri N., Camisa B., Cocchiarella F., Forcato M., Oliveira G., Provasi E., Bondanza A., Bordignon C., Peccatori J., Ciceri F. (2013). IL-7 and IL-15 instruct the generation of human memory stem T cells from naive precursors. Blood.

[29] Peter M.E., Hadji A., Murmann A.E., Brockway S., Putzbach W., Pattanayak A., Ceppi P. (2015). The role of CD95 and CD95 ligand in cancer. Cell Death Differ..

[30] O’Connell J., O’Sullivan G.C., Collins J.K., Shanahan F. (1996). The Fas counterattack: Fas-mediated T cell killing by colon cancer cells expressing Fas ligand. J. Exp. Med..

[31] Schneider D., Xiong Y., Hu P., Wu D., Chen W., Ying T., Zhu Z., Dimitrov D.S., Dropulic B., Orentas R.J. (2018). A unique human immunoglobulin heavy chain variable domain-only CD33 CAR for the treatment of acute myeloid leukemia. Front. Oncol..

[32] Lam N., Trinklein N.D., Buelow B., Patterson G.H., Ojha N., Kochenderfer J.N. (2020). Anti-BCMA chimeric antigen receptors with fully human heavy-chain-only antigen recognition domains. Nat. Commun..

[33] Holliger P., Hudson P.J. (2005). Engineered antibody fragments and the rise of single domains. Nat. Biotechnol..

[34] Kumar M., Keller B., Makalou N., Sutton R.E. (2001). Systematic determination of the packaging limit of lentiviral vectors. Hum. Gene Ther..

[35] Azzam H.S., Grinberg A., Lui K., Shen H., Shores E.W., Love P.E. (1998). CD5 expression is developmentally regulated by T cell receptor (TCR) signals and TCR avidity. J. Exp. Med..

[36] Tarakhovsky A., Müller W., Rajewsky K. (1994). Lymphocyte populations and immune responses in CD5-deficient mice. Eur. J. Immunol..

[37] Alotaibi F., Rytelewski M., Figueredo R., Zareardalan R., Zhang M., Ferguson P.J., Maleki Vareki S., Najajreh Y., El-Hajjar M., Zheng X. (2020). CD5 blockade enhances ex vivo CD8^+^ T cell activation and tumour cell cytotoxicity. Eur. J. Immunol..

[38] James S.E., Greenberg P.D., Jensen M.C., Lin Y., Wang J., Till B.G., Raubitschek A.A., Forman S.J., Press O.W. (2008). Antigen sensitivity of CD22-specific chimeric TCR is modulated by target epitope distance from the cell membrane. J. Immunol..

[39] Xu J., Chen L.-J., Yang S.-S., Sun Y., Wu W., Liu Y.-F., Xu J., Zhuang Y., Zhang W., Weng X.Q. (2019). Exploratory trial of a biepitopic CAR T-targeting B cell maturation antigen in relapsed/refractory multiple myeloma. Proc. Natl. Acad. Sci. USA.

[40] Fan X., Zhuang Q., Wang P., Wang L., Yang L., Hao J., Zhao D., He X. (2020).

[41] Miyazaki K., Yamaguchi M., Suzuki R., Kobayashi Y., Maeshima A.M., Niitsu N., Ennishi D., Tamaru J.I., Ishizawa K., Kashimura M. (2011). CD5-positive diffuse large B-cell lymphoma: a retrospective study in 337 patients treated by chemotherapy with or without rituximab. Ann. Oncol..

[42] Zhao P., Li L., Zhou S., Qiu L., Qian Z., Liu X., Meng B., Zhang H. (2019). CD5 expression correlates with inferior survival and enhances the negative effect of p53 overexpression in diffuse large B-cell lymphoma. Hematol. Oncol..

[43] Wang H.-Y., Zu Y. (2017). Diagnostic algorithm of common mature B-cell lymphomas by immunohistochemistry. Arch. Pathol. Lab. Med..

[44] Zah E., Lin M.Y., Silva-Benedict A., Jensen M.C., Chen Y.Y. (2016). T cells expressing CD19/CD20 bispecific chimeric antigen receptors prevent antigen escape by malignant B cells. Cancer Immunol. Res..

[45] Ruella M., Barrett D.M., Kenderian S.S., Shestova O., Hofmann T.J., Perazzelli J., Klichinsky M., Aikawa V., Nazimuddin F., Kozlowski M. (2016). Dual CD19 and CD123 targeting prevents antigen-loss relapses after CD19-directed immunotherapies. J. Clin. Invest..

[46] Martyniszyn A., Krahl A.C., André M.C., Hombach A.A., Abken H. (2017). CD20-CD19 bispecific CAR T cells for the treatment of B-cell malignancies. Hum. Gene Ther..

[47] De Munter S., Ingels J., Goetgeluk G., Bonte S., Pille M., Weening K., Kerre T., Abken H., Vandekerckhove B. (2018). Nanobody based dual specific CARs. Int. J. Mol. Sci..

[48] Gundry M.C., Brunetti L., Lin A., Mayle A.E., Kitano A., Wagner D., Hsu J.I., Hoegenauer K.A., Rooney C.M., Goodell M.A., Nakada D. (2016). Highly efficient genome editing of murine and human hematopoietic progenitor cells by CRISPR/Cas9. Cell Rep..

[49] Dai Z., Hu X., Jia X., Liu J., Yang Y., Niu P., Hu G., Tan T., Zhou J. (2021). Development and functional characterization of novel fully human anti-CD19 chimeric antigen receptors for T-cell therapy. J. Cell. Physiol..

