# Supplementary material for: The rational development of CD5-targeting biepitopic CARs with fully human heavy-chain-only antigen recognition domains
Source: Mol Ther. 2021 Jul 16;29(9):2707–22. doi: 10.1016/j.ymthe.2021.07.001 (PMC8417515; doi:10.1016/j.ymthe.2021.07.001)
Supplement: Document S1. Figures S1–S7 and Table S1 [file mmc1.pdf]

YMTHE, Volume *29*

Supplemental Information

The rational development of CD5-targeting biepitopic CARs with fully human heavy-chain-only antigen recognition domains

Zhenyu Dai, Wei Mu, Ya Zhao, Xiangyin Jia, Jianwei Liu, Qiaoe Wei, Taochao Tan, and Jianfeng Zhou

A

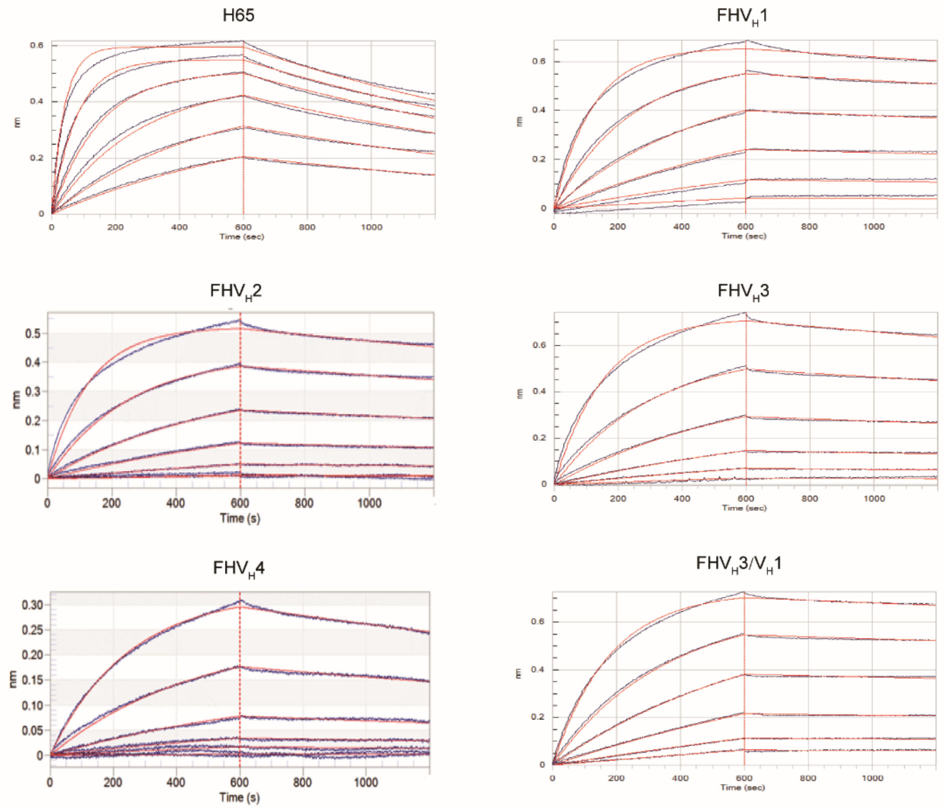

B

Analyte	K_D (M)	K_{on} (1/Ms)	K_{dis} (1/s)
H65	6.53E-10	9.82E+05	6.42E-04
FHV_H1	1.63E-09	8.15E+04	1.33E-04
FHV_H2	8.32E-09	2.55E+04	2.12E-04
FHV_H3	1.39E-09	1.27E+05	1.75E-04
FHV_H4	2.47E-08	1.25E+04	3.09E-04
FHV_H3/V_H1	7.01E-10	1.12E+05	7.83E-05

Supplementary Figure 1. The binding affinity measurement of FHV_H1, FHV_H3, FHV_H4, FHV_H3/V_H1, and H65 antibodies to CD5 antigen. A, The affinity between H65, anti-CD5 V_H domains, biepitopic FHV_H3/V_H1, and recombinant human CD5 was determined using bio-layer interferometry. CD5 antibodies were evaluated in human IgG1 format. **B,** Tabulated kinetic and equilibrium dissociation constants (K_D's) of CD5 antibodies.

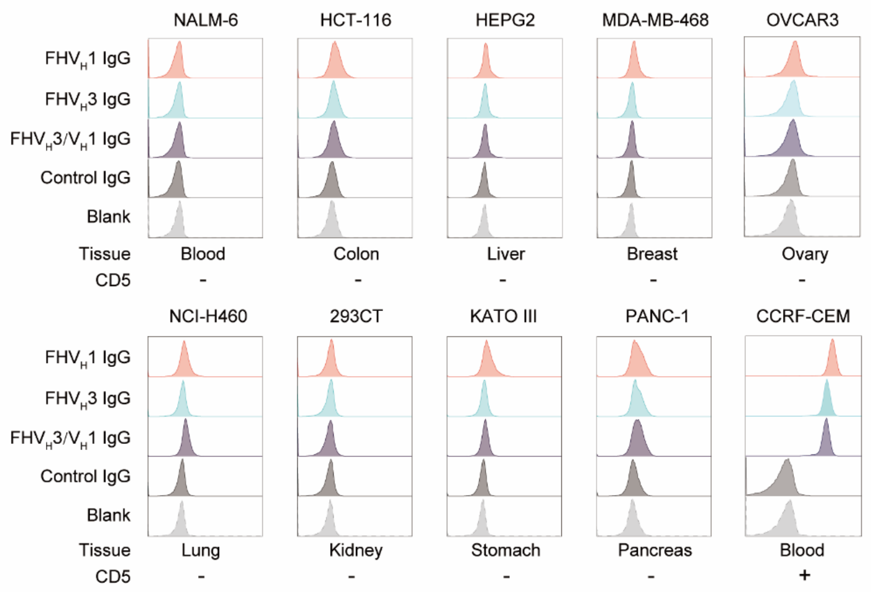

Supplementary Figure 2. Binding test of CD5 antibodies to CD5^{-} cell lines of different tissue origins. CD5^{-} cell lines of different tissue origins and CD5^{+} CCRF-CEM cells as positive control were stained with H65-rFc, FHV$_H$1-rFc, FHV$_H$3-rFc, and FHV$_H$3/V$_H$1-rFc antibodies, followed by PE-conjugated anti-rabbit IgG antibody (clone: Poly4064), then analyzed using flow cytometry.

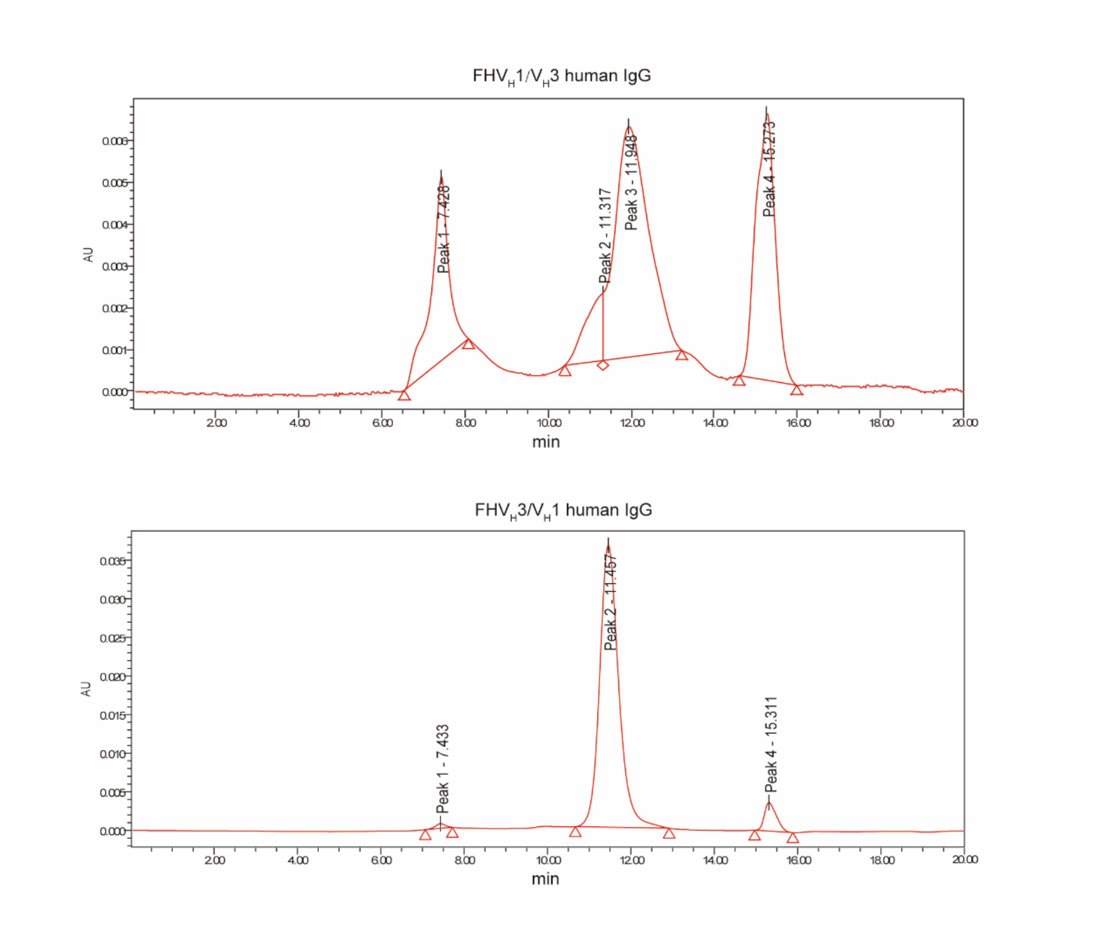

Supplementary Figure 3. Representative size exclusion high-performance liquid chromatography (SEC-HPLC) elution chromatograms of purified CD5 biepitopic antibodies. SEC-HPLC analysis of CD5 biepitopic antibodies, including monomers, aggregates, and fragments, was performed to explore possible reasons for the non-specificity of FHV$_H$1/V$_H$3-hFc antibody.

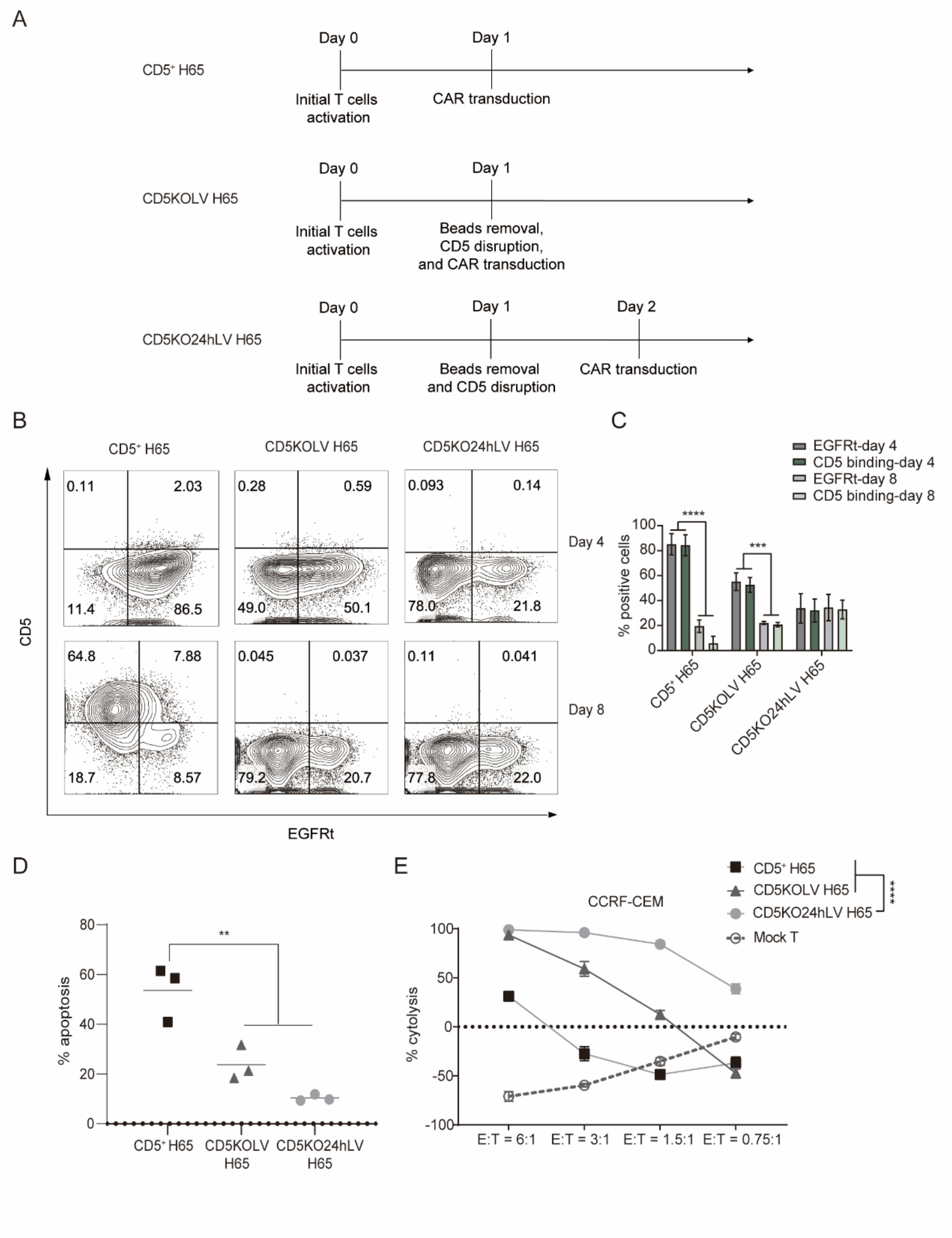

Supplementary Figure 4. Process optimization of CD5KO and lentiviral transduction eliminate fratricide of H65 CAR-T cells. A, Schematic representation of 3 different strategies to generate CD5KO anti-CD5 CAR-T cells. **B,** Representative results showed CD5 expression and EGFR expression of CAR-T cells on days 4 and

8, respectively, after H65 CAR lentivirus transduction through fluorescent-activated cell sorting (FACS). **C,** CAR expression in H65 CAR-T cells measured by EGFR antibody and CD5 antigen on days 4 and 8, respectively. The data represent mean ± SD (n = 3). ***$P < 0.001$, ****$P < 0.0001$ (two-way ANOVA). **D,** Basal apoptosis of $CD5^+$ T cells transduced with H65 CAR lentivirus, T cells transduced with H65 CAR lentivirus immediately after CD5 knockout, and T cells transduced with anti-CD5 CAR lentivirus 24 h after CD5 knockout. Apoptosis was measured using annexin V and PI staining 10 days post-transduction (gated on $EGFR^+$ cells). The data represent mean ± SD (n = 3). **$P < 0.01$ (one-way ANOVA). **E,** Cytotoxicity of CAR-T/T cells to $CD5^+$ CCRF-CEM was determined by luciferase-based cytotoxicity assay after 24 h incubated with target cells at different E:T ratios. The data indicates mean ± SD from three co-cultures. ****$P < 0.0001$ (two-way ANOVA).

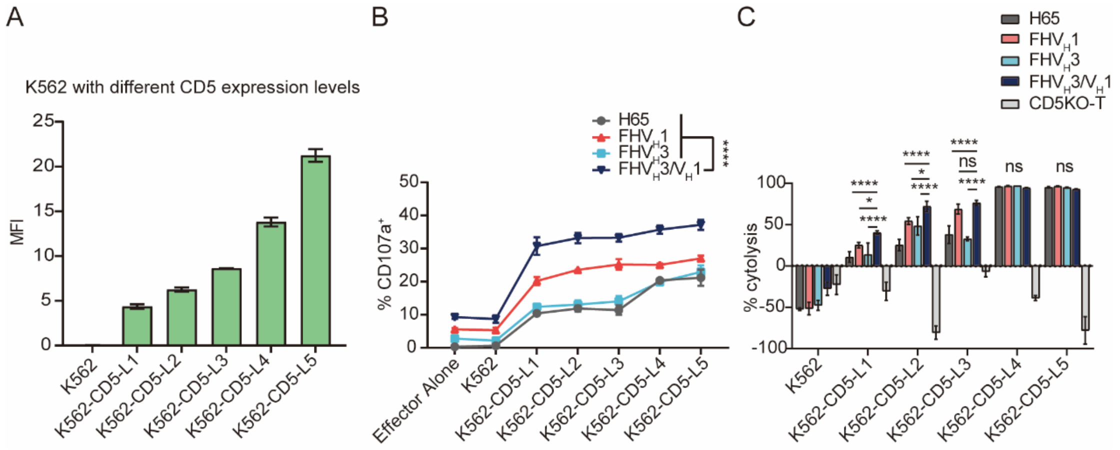

Supplementary Figure 5. Biepitopic FHV$_H$3/V$_H$1 CAR-T cells exhibited higher levels of degranulation and greater cytotoxicity with relatively low levels of CD5 antigen stimulation. A,** Expression of CD5 antigen on the surface of K562 and K562-CD5 L1–5 as determined using flow cytometry after staining with APC-conjugated CD5 antibody. The data represent mean ± SD (n = 3). **B, Degranulation assay of H65, FHV$_H$1, FHV$_H$3, and FHV$_H$3/V$_H$1 CAR-T cells after stimulation by K562 and K562-CD5 L1–5 at an E:T ratio of 10:1 for 4 h (gated on EGFR$^+$CD8$^+$ cells). The data represent mean ± SD (n = 3). ****$P < 0.0001$ (two-way ANOVA). **C,** Cytotoxicity of CAR-T/T cells to K562 and K562-CD5 L1–5 was determined by luciferase-based cytotoxicity assay after 24 h incubated with target cells at an E:T ratio of 3:1. The data indicates mean ± SD from three co-cultures. Not significant (ns), *$P < 0.05$, ****$P < 0.0001$ (two-way ANOVA).

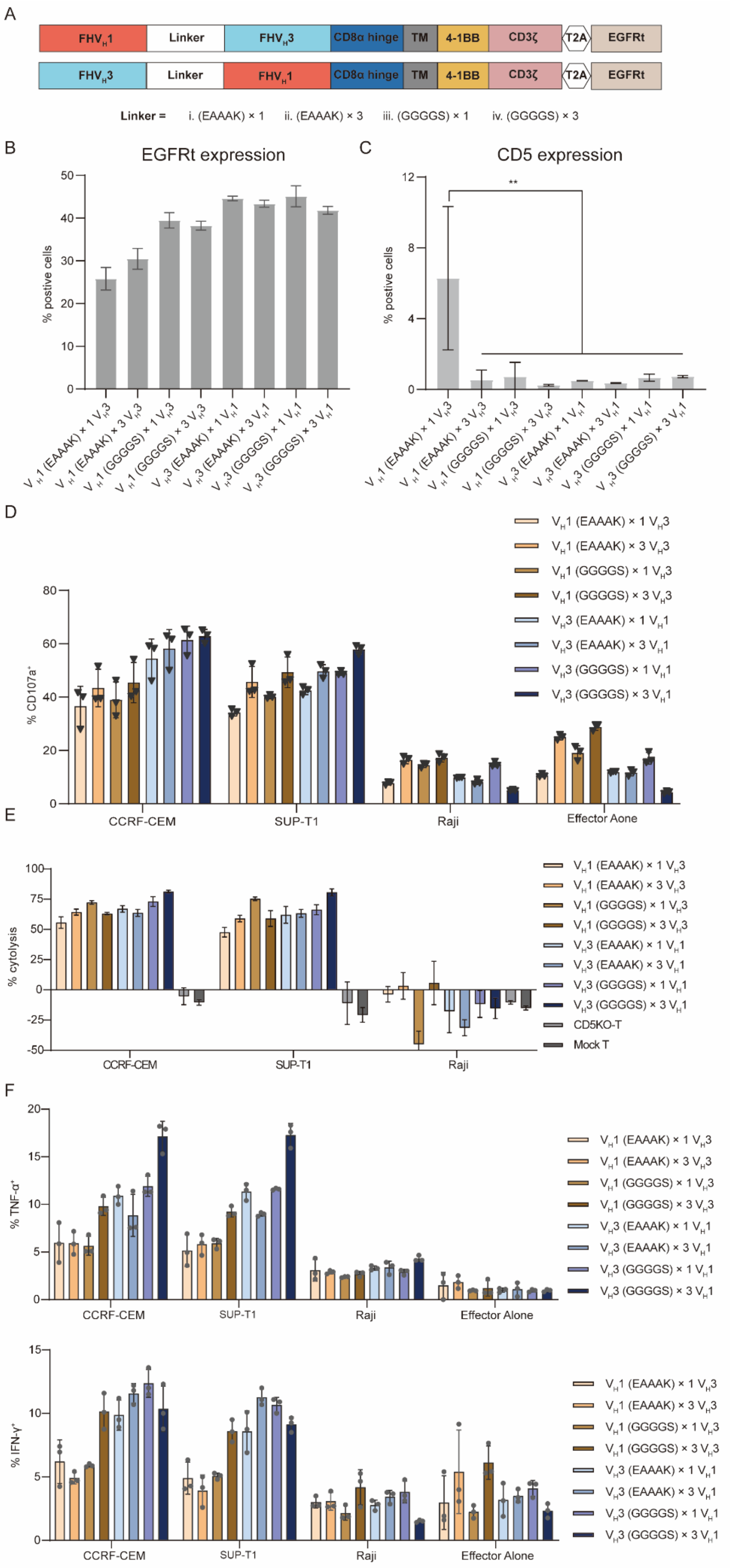
A
FHV_H1
Linker
FHV_H3
CD8α hinge
TM
4-1BB
CD3ζ
T2A
EGFRt
Linker = i. (EAAAK) × 1 ii. (EAAAK) × 3 iii. (GGGGS) × 1 iv. (GGGGS) × 3
B
EGFRt expression
% postive cells
C
CD5 expression
% postive cells
**
V_H1 (EAAAK) × 1 V_H3
V_H1 (EAAAK) × 3 V_H3
V_H1 (GGGGS) × 1 V_H3
V_H1 (GGGGS) × 3 V_H3
V_H3 (EAAAK) × 1 V_H1
V_H3 (EAAAK) × 3 V_H1
V_H3 (GGGGS) × 1 V_H1
V_H3 (GGGGS) × 3 V_H1
D
% CD107a+
CCRF-CEM
SUP-T1
Raji
Effector Aone
E
% cytolysis
CD5KO-T
Mock T
CCRF-CEM
SUP-T1
Raji
F
% TNF-α+
% IFN-γ+
CCRF-CEM
SUP-T1
Raji
Effector Alone

Supplementary Figure 6. Functional comparisons of tandem V_H CARs used either linkers of different sizes, flexibilities, and amino acid compositions or V_H domains connected in different orders (V_H1-V_H3 or V_H3-V_H1). A,** Schematic diagram of the lentiviral vectors encoding the tandem CAR constructs consisting of FHV_H1 and FHV_H3. **B,** The CAR expression levels of tandem CAR constructs measured by EGFR antibody (clone: AY13) on day 10. The results are displayed as mean ± SD (n = 3). **C, Flow cytometry analysis of CD5 antigen expression on the surface of tandem CAR-T cells on day 10. The results are displayed as mean ± SD (n = 3). $^{**}P < 0.01$ (one-way ANOVA). **D,** The degranulation assay of indicated CAR constructs T cells. The results are displayed as mean ± SD (n = 3). **E,** Target-cell lysis activity of tandem CARs determined by luciferase-based cytotoxicity assay after 24 h incubated with target cells at an E:T ratio of 1:1. Reported values are the mean ± SD of triplicates. **F,** Representative flow cytometry analysis showing intracellular cytokine staining for pro-inflammatory cytokines (TNF-α and IFN-γ) after tandem CAR-T cells co-cultured with indicated target cells at an E:T ratio of 1:1 for 5 h. The results are displayed as mean ± SD (n = 3).

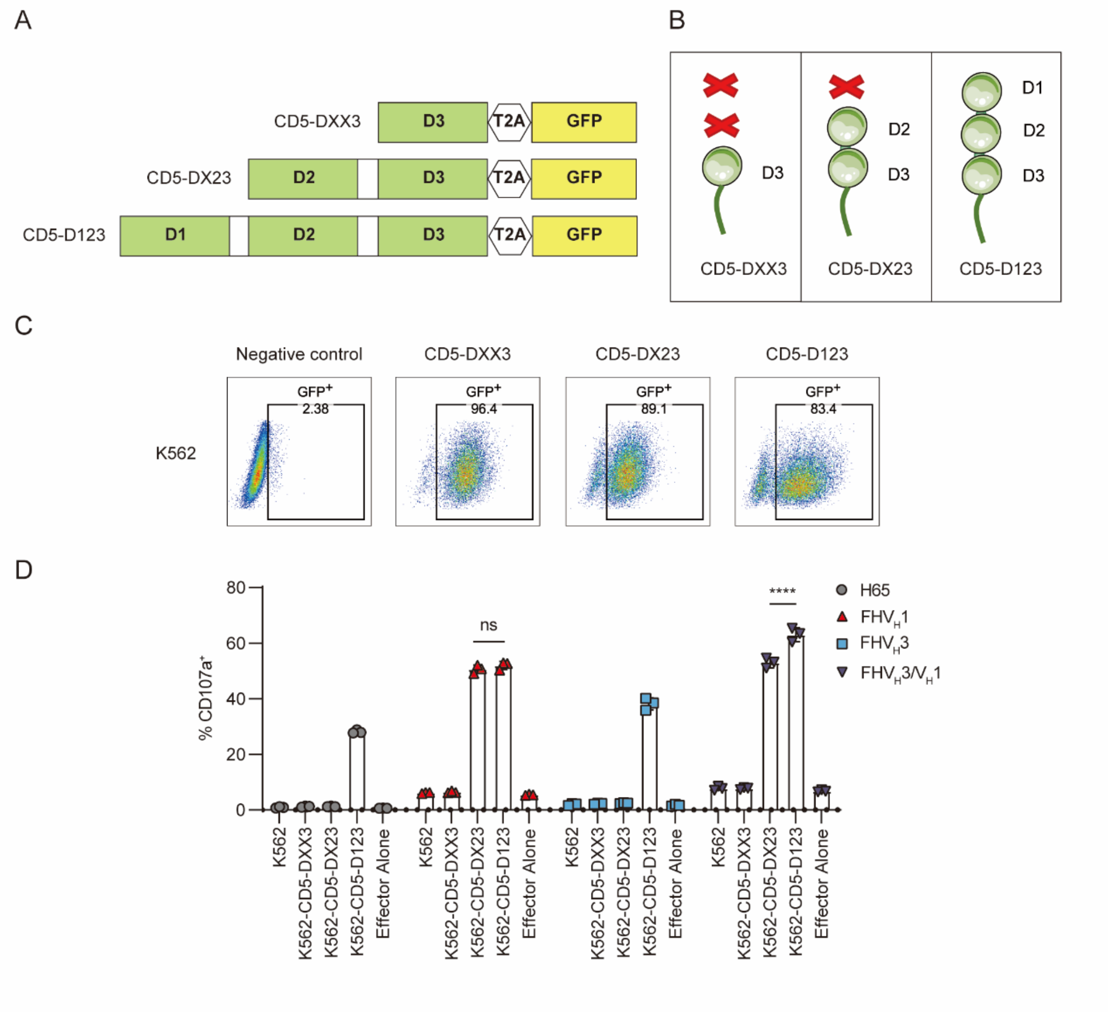

Supplementary Figure 7. Epitope binding specificity of different anti-CD5 CAR constructs. A, Design of vector constructs of wild-type and truncated CD5. The CD5 gene was linked to a GFP by T2A for further detection. **B,** Schematic diagram of wild-type CD5 and CD5 mutants. **C,** Representative results showed GFP expression of K562, K562-CD5-DXX3, K562-CD5-DX23, and K562-CD5-D123. **D,** Degranulation assay of four groups of CAR-T cells stimulated with K562 expressing wild-type and mutated CD5. H65, FHV$_H$1, FHV$_H$3, and FHV$_H$3/V$_H$1 CAR-T cells were incubated with K562, K562-CD5-DXX3, K562-CD5-DX23, and K562-CD5-D123, respectively. CD107a expression levels were detected after 4 h of incubation (gated

on $EGFR^+CD8^+$ cells). The data represents mean ± SD (n = 3). Not significant (ns), ****$P < 0.0001$ (two-way ANOVA).

Supplementary Table 1. Phage protein panning.

Phage V_H antibody library	Round	Strategy	Recovery rates	Enrichment
	1st	Protein	3.34E-05	/
SD-1	2nd	Protein	7.74E-05	2.32
	3rd	Protein	4.54E-03	58.66
	1st	Protein	4.54E-05	/
NV_H	2nd	Protein	7.46E-05	1.64
	3rd	Protein	1.20E-03	16.09
